# Linear Regression QSAR Models for Polo-Like Kinase-1 Inhibitors

**DOI:** 10.3390/cells7020013

**Published:** 2018-02-14

**Authors:** Pablo R. Duchowicz

**Affiliations:** Instituto de Investigaciones Fisicoquímicas Teóricas y Aplicadas (INIFTA), CONICET, UNLP, Diag. 113 y 64, C.C. 16, Sucursal 4, La Plata 1900, Argentina; pabloducho@gmail.com; Tel.: +54-(221)-425-7430; Fax: +54-(221)-425-4642

**Keywords:** polo-like kinase-1 inhibitors, quantitative structure-activity relationships, half-maximal inhibitory concentration, replacement method, molecular descriptors

## Abstract

A structurally diverse dataset of 530 polo-like kinase-1 (PLK1) inhibitors is compiled from the ChEMBL database and studied by means of a conformation-independent quantitative structure-activity relationship (QSAR) approach. A large number (26,761) of molecular descriptors are explored with the main intention of capturing the most relevant structural characteristics affecting the bioactivity. The structural descriptors are derived with different freeware, such as PaDEL, Mold^2^, and QuBiLs-MAS; such descriptor software complements each other and improves the QSAR results. The best multivariable linear regression models are found with the replacement method variable subset selection technique. The balanced subsets method partitions the dataset into training, validation, and test sets. It is found that the proposed linear QSAR model improves previously reported models by leading to a simpler alternative structure-activity relationship.

## 1. Introduction

Polo-like kinases (PLKs) are characterized by a multidomain structure consisting of a highly conserved N-terminal catalytic domain (KD) and a relatively divergent C-terminal polo-box domain (PBD), composed of either one or two polo boxes [1,2]. This serine/threonine kinase family is an important regulator of mitotic progression [3]. 

Among the different identified PLKs, PLK1 is the most investigated member of the family because it is highly expressed in proliferating cells and overexpressed in many cancers, thus resulting in an attractive target for anticancer therapeutic development [4].

Polo-like kinase 1 (PLK1) is involved in centrosome maturation, kinetochore function, spindle formation, chromosome segregation, and cytokinesis [5]. The PBD is critical to PLK1 localization and function and negatively regulates the kinase activity of the catalytic domain [1]. Inhibitors targeting KD, the so-called ATP-competitive PLK1 inhibitors, have attracted much attention over the last years [6]. 

**Figure 1:**
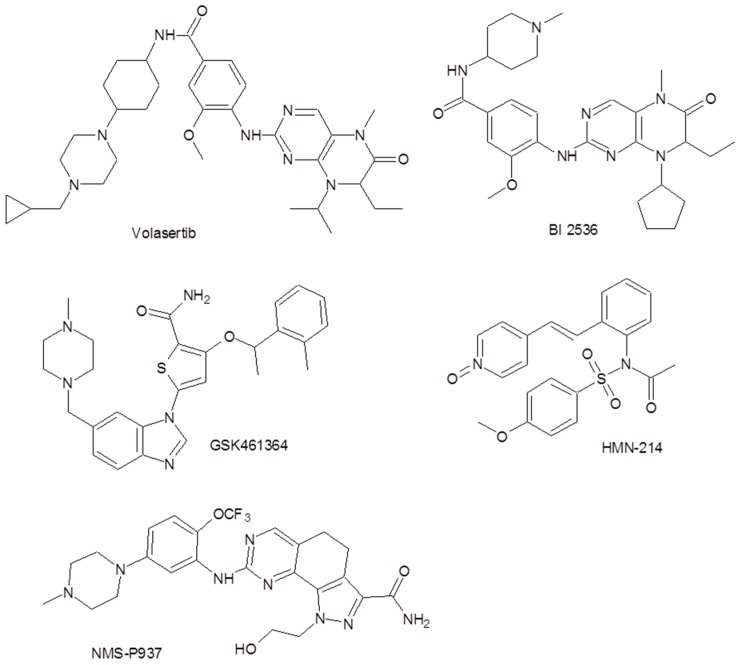
Some polo-like kinase-1 (PLK1) inhibitors involved in current clinical trials [5].

Inhibiting PLK1 activity results in a potent antitumor effect both in vitro and in vivo [7]; therefore, much attention has been focused on characterizing PLK1 and synthesizing its inhibitors [5]. Figure 1 shows the molecular structures of some encouraging agents in current clinical trials. 

Among the various methodologies available in the literature for predicting the biological activities of compounds, the quantitative structure-activity relationship (QSAR) theory [8,9,10,11,12] is considered as a useful and well-known strategy. The main hypothesis behind every QSAR study is that the chemical structure is responsible for the bioactivity of the compound. 

Therefore, the structure is quantified through molecular descriptors; in other words, numerical quantities carrying specific and relevant information about the constitutional, topological, geometrical, hydrophobic, and/or electronic characteristics of the compounds are investigated [13,14,15,16]. Thousands of molecular descriptors are now available in the literature, and it has to be decided how to select those numerical variables that best characterize the experimental activity under consideration. 

The most relevant descriptors are selected with an appropriate mathematical technique and statistically correlated to the experimental activity, resulting in a mathematical model that is used to find out useful structure-activity parallelisms. In this way, QSAR models constitute a fast and cost-effective alternative to experimental measurements. 

The availability of newer and higher quality experimental measurements has encouraged and justified the development of newer and alternative QSAR models with improved statistical quality; therefore, this research field has continued to evolve over the years.

A large number of structure-activity relationship (SAR) studies has been reported in the past for analyzing the PLK1 inhibition although, however, very few account for quantitative approaches [17,18,19,20]. Therefore, in the present work, we develop a QSAR analysis for searching predictive models on a large and structurally diverse dataset of 530 PLK1 inhibitors. For this purpose, we resort to the conformation-independent QSAR approach and consider constitutional and topological representations of the inhibitors’ chemical structures for deriving the molecular descriptors.

The main advantage of not considering molecular conformations is that the only experimental data needed for establishing the QSAR models is the experimental inhibitory activity being analyzed [21,22,23,24]. No further experimental information is required, such as data on the experimental X-ray crystal structure of the PLK1 kinase domain with a given inhibitor in certain conformation [25]. In addition, it is known that the ligand–receptor complex has not been solved for all inhibitor types having different interaction modes—that is, in a heterogeneous dataset like the one considered in this work. 

The inclusion of more specific experimental information is appropriate whenever a microscopic and more sophisticated modeling methodology is involved, such as CoMFA (comparative molecular field analysis) and CoMSIA (comparative molecular similarity indices analysis) [17,26]. However, as commented previously, such specific experimental details are usually unavailable for any chemical system of interest; therefore, the application of the conformation-independent QSAR approach can be considered as a useful and valid alternative.

## 2. Materials and Methods

### 2.1. Experimental Dataset

The structurally diverse PLK1 inhibitors were compiled from ChEMBL [27,28], an open data resource of binding, functional, and ADMET bioactivity data. The experimental inhibitory effectiveness is expressed as the half-maximal inhibitory concentration $IC_{50}$ (nM). 

After removing duplicates, compounds with ambiguous data, compounds having molecular weights higher than 1000 g mol^−1^, and compounds without reported bioactivities, the dataset consisted of 530 compounds with $IC_{50}$ values ranging from 0.8 to 145,000 nM and molecular weights ranging from 164.2 to 949.97 g mol^−1^. The complete list of compounds studied here is provided in Table S1 as Supplementary Material.

### 2.2. Structural Representation and Molecular Descriptors Calculation

The 530 chemical structures studied here are provided as canonical SMILES notation in Table S1 of the Supplementary Material section. All file format conversions were performed with Open Babel for Windows [29]. The molecular structures were visualized with ACDLabs ChemSketch freeware [30]. 

The conformation-independent molecular descriptors were computed as follows. We used the Pharmaceutical Data Exploration Laboratory (PaDEL) freeware version 2.20 [31] because it has the advantage that it is a freely available and open-source program. PaDEL allowed us to calculate 1444 0D-2D descriptors and 12 fingerprint types (16,092 bits) [32]. The categorical (indicator) fingerprint descriptors involve the presence or count of specific chemical substructures: we treated the fingerprints like they were “constitutional descriptors” describing the molecular composition and, as such, they could be used for modeling any property of interest.

More molecular descriptors were calculated with the Molecular Descriptors from 2D structures (Mold^2^) freeware [33], which generated 777 1D-2D structural variables with molecules in MDL sdf format. 

Finally, 2D molecular descriptors were calculated with the Quadratic, Bilinear and N-Linear MapS (QuBiLs) [34] suite by using the graph-theoretic electronic-density matrices and atomic weightings (MAS) module from the ToMoCoMD-CARDD free multi-platform freeware. The QuBiLs-MAS algebraic module calculated 8448 quadratic, bilinear, and linear maps based on pseudograph-theoretic electronic-density matrices and atomic weightings, when the program was used with the following options selected: ‘bilinear’, ‘linear’, and ‘quadratic’ algebraic forms; ‘atom-based’, ‘non-chiral’ and ‘duplex’ constraints; ‘non-stochastic’, ‘simple stochastic’, ‘double stochastic’ and ‘mutual probability’ matrix forms (maximum order 15); ‘keep all’ cut-off; ‘total’ groups; ‘Ghose-Crippen LogP’, ‘polarizability’, ‘charge’, ‘polar surface area’, ‘electronegativity’, ‘refractivity’, ‘mass’ and ‘van der Waals volume’ properties; ‘Euclidean distance’, ‘arithmetic mean’, and ‘standard deviation’ invariants (non-standardized option). 

Through PaDEL, Mold^2^, and QuBiLs-MAS we derived 26,761 non-conformational molecular descriptors with the intention of exploring the most relevant structural characteristics affecting the studied PLK1 bioactivity.

### 2.3. Model Development 

#### 2.3.1. Molecular Descriptors Selection

First, the ‘collinear’ or linearly dependent descriptor pairs were identified, and only one variable from each pair was kept for further analysis. Non-informative descriptors not relevant to the QSAR analysis were excluded (i.e., descriptors with constant and near-constant values and descriptors with at least one missing value), leading to a pool of 11,565 linearly independent non-conformational descriptors.

We employed the replacement method (RM) technique [35] in order to generate multivariable linear regression (MLR) models on the training set (train) by searching in a pool having *D* = 11,565 descriptors for optimal subsets containing *d* descriptors (*d* is much lower than *D*), with the smallest values for the standard deviation ($S_{train}$). 

The main idea behind the RM is that one can approach the minimum of $S_{train}$ by judiciously taking into account the relative errors of the coefficients of the least-squares model given by a set of *d* descriptors. In other words, we should find the global minimum of $S_{train}(d)$ in a subspace of D!/[d!(D−d)!] points *d*, where *D* represents the total number of available descriptors. 

The quality of the results achieved with this technique approaches that obtained by performing an exact (combinatorial) full search of molecular descriptors although, of course, requires much less computational work. The RM is computationally more expensive than the stepwise regression (SR) and genetics algorithm (GA) approaches, although produces similar or better results than GA and better results than SR [36].

Table S2 includes a list of mathematical equations involved in the present study. All the MatLab programmed algorithms used in our calculations are available upon request.

#### 2.3.2. Model Validation

The complete molecular set of 530 inhibitors was split into three subsets: training (*train*), validation (*val*), and test sets. The training set was used to calibrate the model and to obtain its parameters through the RM technique, while the validation set helped to calibrate and partially validate the model by predicting the bioactivity of compounds not included in train. Finally, the test set contained compounds “never seen” during the calibration step with *train* and *val*, and demonstrates the real predictive capability of the QSAR.

The dataset partitioning has to achieve similar structure-activity relationships in the three subsets; in other words, the training set molecules should be representative of the validation and test set compounds. For this purpose, the split of the dataset was carried out by means of the balanced subsets method (BSM) [37,38], a procedure proposed by our group that ensures that balanced subsets are generated. The BSM is based on the k-means cluster analysis (k-MCA) method [39]: the essence of k-MCA is to create k-clusters or groups of compounds in such a way that compounds in the same cluster are very similar in terms of distance metrics (i.e., Euclidean distance), and compounds in different clusters are very distinct. 

The linear regression models are also theoretically validated through the leave-one-out cross-validation (*loo*) procedure [40], and also through the more rigorous leave-30%-out cross-validation (l30‰) method.

#### 2.3.3. Applicability Domain

A predictive QSAR model is only able to predict molecules falling within its applicability domain (AD), so that the predicted activity is not a result of substantial extrapolation (unreliable prediction) [41,42]. The AD definition is dependent on the model’s descriptors and the experimental activity.

In this work, we determined the AD through two alternative methodologies. The first one is based on the well-known leverage approach [43] where a test set compound *i* must have a calculated leverage $h_{i}$ smaller than the warning leverage $h^{*}$. The second one is based on a simple standardization approach [42]: a given test set compound *i* having *d* standardized descriptor values $s_{ik},k=1,\ldots ,d$ must have a maximum value $s_{ik}^{\max}\leq 3$. In the case that $s_{ik}^{\max}>3$ and its minimum value $s_{ik}^{\min}<3$, then the $s_{i}^{new}$ parameter has to be calculated and must fulfill the condition: $s_{i}^{new}=\langle s_{i}\rangle +1.28.\sigma_{s_{i}}\leq 3$, where $\langle s_{i}\rangle$ is the mean of $s_{ik}$ values for *i* and $\sigma_{s_{i}}$ is the standard deviation for such values.

## 3. Results and Discussion

After partitioning the dataset of 530 PLK1 inhibitors into *train*, *val*, and test sets using the BSM technique, we obtain balanced subsets with $N_{train}=265$, $N_{val}=133$ and $N_{test}=132$ compounds; in addition, Table S1 denotes the members of *val* (^) and *test* (*) sets. Therefore, the calibration compounds in train and *val* constitute 75% of the whole dataset. 

The best MLR models, including the most representative 1–9 molecular descriptors, are presented in Table 1. A brief description of such descriptors is also supplied in Table S3. From the results of Table 1, it is clearly appreciated that the $S_{train}$ parameter continuously improves with the addition of molecular descriptors into the linear equation. However, according to the validation set results, the most predictive models (lowest $S_{val}$) have 8 and 9 descriptors. We kept the model’s dimension as small as possible and selected the following 8-descriptor model and associated statistical quality:

(1) $$\begin{array}{l} \log_{10}IC_{50}=0.46mindssC-0.85maxHCsats+0.88M66-0.54PC494-\\ \;2.76PC534-1.12PC686+2.68KR3577-1.44KR4268+4.37 \end{array}$$

$$N_{train}=265,\;R_{train}^{2}=0.69,\;S_{train}=\;0.80$$

$$R_{ij}^{2\max}=0.14,\;VIF^{\max}=1.11,\;o3=1,\;R_{rand}^{2}=0.13,\;S^{rand}=1.35$$

$$R_{loo}^{2}=0.67,\;S_{loo}=0.83,\;R_{l30\%o}^{2}=0.58,\;S_{l30\%o}=0.95$$

$$N_{val}=133,\;R_{val}^{2}=0.75,\;S_{val}=0.82$$

$$N_{test}=132,\;R_{test}^{2}=0.69,\;S_{test}=0.85$$

**Figure 2:**
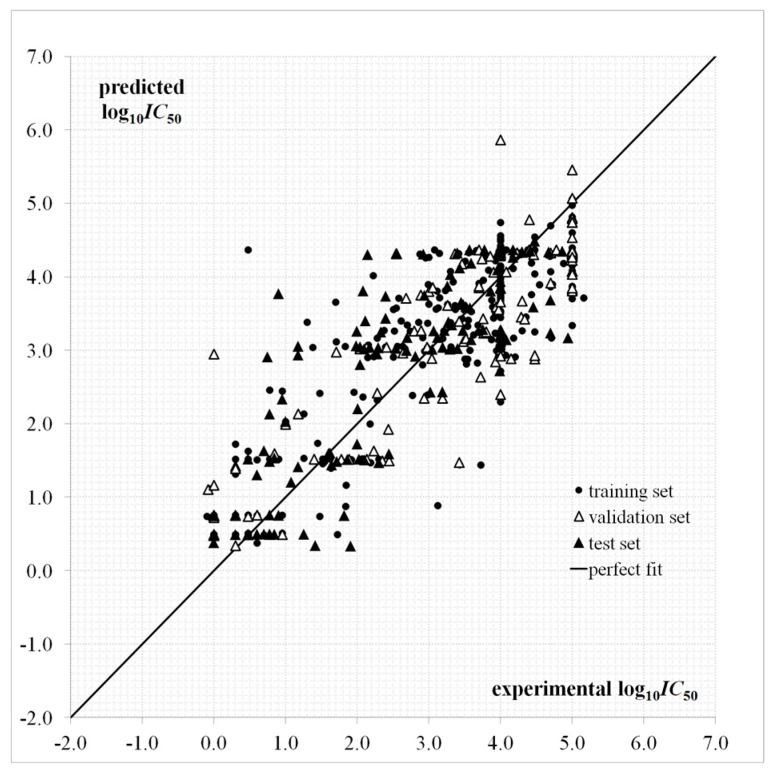
Predicted and experimental $\log_{10}IC_{50}$ values according to the quantitative structure-activity relationship (QSAR) of Equation (1).

**Figure 3:**
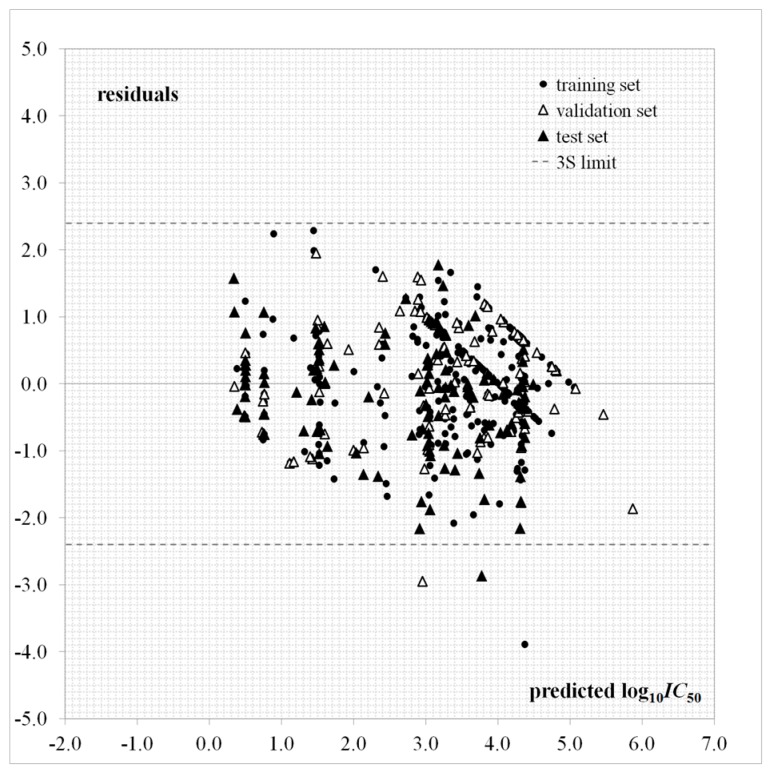
Dispersion plot of residuals for Equation (1).

A plot for the $\log_{10}IC_{50}$ predictions given by Equation (1) as a function of the experimental values is provided in Figure 2. The dispersion plot of residuals in Figure 3 tends to obey a random pattern around the zero line, suggesting that Equation (1) predicts the whole dataset without systematic errors or residual bias. 

The o3 parameter indicates the number of outlier compounds in the training set having a residual (difference between experimental and predicted activity) greater than 3 times $S_{train}$. The only outlier in the training set is **171**, 1-{4-[(4-chlorophenyl)methoxy]-3-methoxyphenyl}-*N*-[(pyridin-4-yl)methyl]methanamine. After close inspection of this specific compound, it is easily concluded that the abnormal behavior can be completely attributed to the highly heterogeneous dataset being analyzed, involving molecular weights from 164.2 to 949.97 g mol^−1^ and bioactivities from 0.8 to 145,000 nM.

Our proposed 8-descriptor model approves the internal validation process of loo and l30‰ (500,000 cases) cross-validation procedures through the prediction of 1 or 80 molecules excluded at a time from the training set. According to the specialized literature [40], the cross-validation $R_{loo}^{2}$ and $R_{l30‰}^{2}$ explained variances should be greater than 0.5, although this is a necessary but not sufficient condition for the real predictive power.

As a way of demonstrating that the QSAR model is not a result of chance correlation, the experimental $\log_{10}IC_{50}$ activity values were scrambled with Y-randomization [44] (100,000 cases). When $S^{rand}$ (*S* for Y-randomization) is greater than $S_{train}$, a valid and useful structure-activity relationship is found, as is the case for Equation (1). 

The recommended external validation criteria to assure predictive capability [40] are also achieved: $1-R_{0}^{2}/R_{test}^{2}(1.18\times 10^{-3})<0.1$ or $1-R_{0}^{\prime 2}/R_{test}^{2}(0.12)<0.1$; 0.85≤k(0.93)≤1.15 or $0.85\leq k^{\prime }(1.00)\leq 1.15$; $R_{m}^{2}(0.67)>0.5$.

The $R_{ij}^{\max}$ parameter from Equation (1) is the maximum correlation coefficient between descriptor pairs: $R_{ij}^{2\max}=0.14$ indicates that there is no serious overlapping structural information. $VIF^{\max}$ is the maximum variance inflation factor, a parameter that measures the maximum multicollinearity among descriptors. A *VIF* of 1 for a specific descriptor means that there is no correlation between this descriptor and all the remaining descriptors of the model, and a *VIF* exceeding 10 indicates that multicollinearity is a problem in the dataset [45]. For Equation (1), $VIF^{\max}=1.11$. The complete squared correlation matrix and *VIF* values are provided in Table S4.

It is known that a successful QSAR model is established only when it surpasses the validation process, in other words, by testing its ability to predict the experimental bioactivity of compounds that are not considered during the model calibration [46,47]. The QSAR of Equation (1) has an acceptable predictive capability for the external test set of 132 “never seen” experimental $\log_{10}IC_{50}$ values according to $R_{test}^{2}$ and $S_{test}$ parameters and Figure 2 and Figure 3. This QSAR can thus be applied to predict new inhibitors with unknown experimental $IC_{50}$.

The eight conformation-independent structural indices of Equation (1) have quite a straightforward structural interpretation:

- Two electrotopological state atom-type descriptors: *mindssC*, the minimum atom-type E-state: =C<; and *maxHCsats*, the maximum atom-type H E-state: H bonded to B, Si, P, Ge, As, Se, Sn, or Pb.
- A MACCS fingerprint descriptor: *M*66, the number of CC(C)(C)A fragments, where A is any valid periodic table element symbol.
- Three PubChem fingerprint descriptors: *PC*494, the presence of O=C-C:N fragment, where ‘:’ denotes bond aromaticity; *PC*534, the presence of S-C:C-O fragment; and *PC*686, the presence of O=C-C-C-C-O fragment.
- Two Klekota–Roth fingerprint descriptors: *KR*3577, the presence of SMARTS substructure Cc1cccc(C)c1NC=O; and *KR*4268, the presence of SMARTS substructure Nc1ccccc1O.

The numerical values for these descriptors are provided in Table S5: all of them have positive numerical values with the exception of *mindssC*, which has either positive or negative values. The sign of the regression coefficient in the linear model indicates when the descriptor contribution increases or decreases the predicted $\log_{10}IC_{50}$ values. Therefore, it is possible to propose the following useful QSAR guide for the chemical synthesis of new PLK1 inhibitors. Molecular structures of inhibitors simultaneously having higher positive values of maxHCsats, PC494, PC534, PC686, and KR4268 and lower values for mindssC, M66, and KR3577 would exhibit lower predicted $\log_{10}IC_{50}$ values, being predicted as more active PLK1 inhibitors.

**Figure 4:**
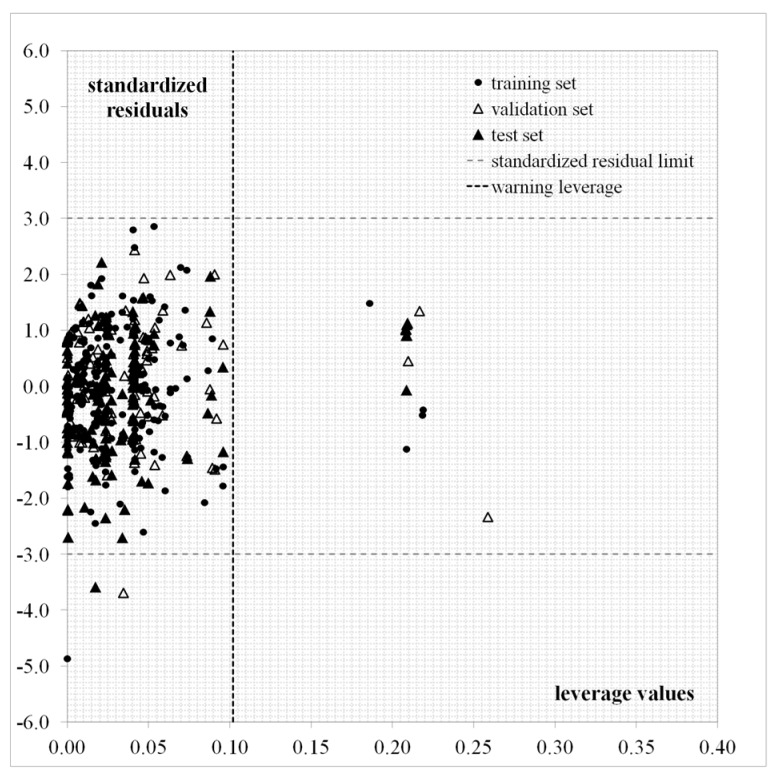
Williams plot for Equation (1).

In order to apply the proposed QSAR guide, the molecular structures to be predicted have to fall within the model’s applicability domain (AD). Within the leverage approach [43], a compound with high leverage ($h_{i}$) would reinforce the model if the compound is in the *train* or *val* (good leverage) calibration sets; but such a compound in the test set could have unreliable predicted data, the result of substantial extrapolation of the model (bad leverage) [41]. Equation (1) reveals that most of the test set compounds have $h_{i}$ values falling under the $h^{*}$ limit (0.1019) with the exception of five test set compounds: **495**, **508**, **509**, **511**, and **516**. The Williams plot (standardized residuals as a function of the $h_{i}$ values) is provided in Figure 4. 

This result obtained with the leverage approach for the test set approximately coincides with the one obtained by using the standardization approach, as the two conditions $s_{ik}^{\max}\leq 3$ or $s_{i}^{new}\leq 3$ are followed by all the test set compounds with the exception of seven compounds: the five previous test compounds and two more compounds lying near the $h^{*}$ limit: **453** and **457**. Thus, the predicted $\log_{10}IC_{50}$ values for most of the test set compounds can be considered as reliable. 

Finally, the obtained regression model in Equation (1) can be converted into a classification model by classifying compounds with experimental *IC*_50_ ≤ 1000 nM as highly active inhibitors and experimental *IC*_50_ > 1000 nM as poorly active inhibitors. Then, the Cooper statistics [48] related to accuracy (*A*%), sensitivity (*SE*), and specificity (*SP*) and the Matthews correlation coefficient (*MCC*) can be calculated. The classification results for Equation (1) in the test set are acceptable as *A*% = 83%, *SE* = 0.73, *SP* = 0.95, *MCC* = 0.69.

A previous study developed by Kong and Yan [20] employed the current ChEMBL database of PLK1 inhibitors for establishing various in silico classification models. The 16 single classifier models and one consensus Kohonen’s self-organizing map (SOM) model were applied to a dataset of 601 noncongeneric PLK1 inhibitors. For these 16 single classifier models, four machine learning methods were used: support vector machine (SVM), naive Bayes (NB), C4.5 decision tree (C4.5 DT), and random forest (RF), with *MCC* ranging from 0.609 to 0.864 and *A*% ranging from 78.7% to 93.1% for the test set. Then, a consensus SOM model was built based on four single classifier models to obtain a more reliable and robust model, outperforming all the single classifier models with *MCC* = 0.87 and *A*% = 93.6% on the test set.

The models reported in [20] achieved acceptable results. However, the linear QSAR model of Equation (1) represents an improved alternative model having the following characteristics:

i. Our proposed model performs both regression and classification.
ii. Dataset partitioning: three subsets are considered, such as *train*, *val*, and test instead of only two (*train* and *test*) in [20]. In this way, it is more convenient for analyzing the predictive performance of the model.
iii. Model’s size: a fewer number of molecular descriptors are involved in the final selected model—i.e., 8 instead of 10–15. Therefore, the parsimony´s principle is accomplished (Ockham’s razor) [49] by following the common practice of keeping the model’s dimension as small as possible.
iv. No energy or geometry optimization is performed on the inhibitor chemical structures. The conformation-independent QSAR approach considers only constitutional and topological representations for deriving the molecular descriptors.
v. A simpler modeling methodology based on MLR analysis is applied in the present study.

## 4. Conclusions

Polo-like kinase-1 is an attractive target for anticancer therapeutic development so the prediction of its inhibitors has been of great interest during the last years. The linear regression QSAR model established in this work on a structurally diverse set of 530 PLK1 inhibitors has an acceptable predictive capability in the external test set and is based on eight non-conformational molecular descriptors. 

For chemical structures falling within the applicability domain of this model, a QSAR guide for the chemical synthesis of new PLK1 inhibitors is provided as follows: molecular structures of inhibitors simultaneously having higher positive values of *maxHCsats*, *PC*494, *PC*534, *PC*686, and *KR*4268 and lower values for *mindssC*, *M*66, and *KR*3577 would exhibit lower predicted log_10_*IC*_50_ values, being predicted as more active PLK1 inhibitors.

The consideration of the constitutional and topological aspects of the molecular structures in the conformation-independent QSAR approach achieves acceptable results. New investigations on other physicochemical and biological properties of interest will be published soon elsewhere.

## Figures and Tables

**Table 1:** Molecular descriptors involved in the best linear regression quantitative structure-activity relationship (QSAR) models for polo-like kinase-1 (PLK1) inhibitors. The selected model appears in bold.

*d*	Descriptors	$R_{train}^{2}$	$S_{train}$	$R_{val}^{2}$	$S_{val}$	$R_{test}^{2}$	$S_{test}$
1	Sub99	0.31	1.18	0.39	1.25	0.28	1.31
2	PC534; AP170	0.49	1.02	0.56	1.08	0.52	1.06
3	PC534; KR4261; AP170	0.52	0.99	0.68	0.95	0.57	0.98
4	nHBAcc3; PC534; KR4261; AP170	0.57	0.94	0.71	0.90	0.62	0.93
5	PC534; KR3577; KR4268; AP170; KRC3897	0.61	0.90	0.71	0.89	0.71	0.83
6	maxHCsats; M66; PC534; KR3577; KR4268; KRC3897	0.64	0.87	0.74	0.85	0.69	0.84
7	maxHCsats; M66; PC534; PC686; KR3577; KR4268; AP159	0.66	0.84	0.74	0.84	0.66	0.89
**8**	**mindssC; maxHCsats; M66; PC494; PC534; PC686; KR3577; KR4268**	**0.69**	**0.80**	**0.75**	**0.82**	**0.69**	**0.85**
9	mindssC; maxHCsats; M66; PC494; PC534; PC686; KR3577; KR4268; APC510	0.70	0.79	0.75	0.82	0.70	0.85

## Supplementary Materials

The following are available online at http://www.mdpi.com/2073-4409/7/2/13/s1.
