# Supplementary material for: Linear Regression QSAR Models for Polo-Like Kinase-1 Inhibitors
Source: Cells. 2018 Feb 14;7(2):13. doi: 10.3390/cells7020013 (PMC5850101; doi:10.3390/cells7020013)
Supplement: Supplementary file 1 [file cells-07-00013-s001.pdf]

Linear Regression QSAR Models for Polo Like Kinase-1 Inhibitors

Pablo R. Duchowicz

Instituto de Investigaciones Fisicoquímicas Teóricas y Aplicadas (INIFTA), CONICET, UNLP, Diag. 113 y 64, C.C. 16, Sucursal 4, 1900 La Plata, Argentina.

Corresponding author. Telephone: (+54)(221)4257430 Fax: (+54)(221)4254642. E-mail: pabloducho@gmail.com

Supporting Information

Table 1S. Experimental (exp.) and predicted (pred., Eq. 1) $\log_{10} IC_{50}$ values for 530 heterogeneous PLK1 inhibitors.

Table 2S. List of mathematical equations used in the present study

Table 3S. A brief description for molecular descriptors appearing in this QSAR study.

Table 4S. Squared correlation matrix for Eq. 1 and the variance inflation factor (*VIF*) for each descriptor.

Table 5S. Numerical descriptor values involved in Eq. 1.

Table 1S. Experimental (exp.) and predicted (pred., Eq. 1) $\log_{10} IC_{50}$ values for 530 heterogeneous PLK1 inhibitors.

ID	ChEMBL ID [28]	molecular weight ($g.mol^{-1}$)	canonical smiles	exp.	pred. Eq. 1
1	CHEMBL1672002	164.2	CC(C)C1=CC(=O)C(=CC1=O)C	3.3385	3.9298
2	CHEMBL3104854	196.16	Cc1c(O)c(O)c(O)c2C(=O)OCc12	4.0719	2.9315
3	CHEMBL1933575	203.2	NC(=O)c1c[nH]c(c1)c2ccnc(N)n2	4.0000	4.1394
4	CHEMBL3104853	212.2	COCc1c(C)c(O)c(O)c(O)c1C=O	4.4771	3.2496
5	CHEMBL225519	213.24	O=C1NCCc2[nH]c(cc12)c3ccncc3	2.9912	3.8937
6^ [a]	CHEMBL3104852	226.18	COC1OC(=O)c2c(O)c(O)c(O)c(C)c12	4.4771	2.8806
7^	CHEMBL402272	229.24	Nc1nccc(n1)c2cc3C(=O)NCCc3[nH]2	3.0535	3.8530
8	CHEMBL313578	237.25	Oc1ccc(\C=C\2/C(=O)Nc3ccccc23)cc1	4.3979	4.3222
9	CHEMBL2407906	237.25	Oc1cccc(\C=C\2/C(=O)Nc3ccccc23)c1	4.3979	4.3137
10^	CHEMBL2407907	237.25	Oc1ccccc1\C=C\2/C(=O)Nc3ccccc23	4.4624	4.3007
11	CHEMBL262623	239.28	Nc1ncnc2NCCC(=Nc12)c3ccccc3	5.0000	4.3199
12^	CHEMBL590769	250.26	O=C1Cc2cnc3ccnn3c2c4ccccc4N1	5.0000	4.3524
13	CHEMBL591896	251.24	O=C1Cc2cnc3ccnn3c2c4ncccc4N1	5.0000	4.3338
14^	CHEMBL608427	252.23	O=C1Cc2cnc3ncnn3c2c4ncccc4N1	5.0000	4.3243
15^	CHEMBL263902	253.3	Cc1cccc(c1)C2=Nc3c(N)ncnc3NCC2	4.6990	4.3234
16^	CHEMBL405233	253.3	Cc1ccc(cc1)C2=Nc3c(N)ncnc3NCC2	5.0000	4.3218
17^	CHEMBL3361128	254.24	Oc1ccc(\C=C/2\Oc3ccccc3C2=O)cc1O	5.0000	4.2765
18^	CHEMBL259380	254.29	Nc1nc(N)c2N=C(CCNc2n1)c3ccccc3	5.0000	4.2911
19	CHEMBL466154	258.23	Cc1cc(O)cc2Oc3cc(O)cc(O)c3C(=O)c12	4.8791	4.1810
20* [b]	CHEMBL2407910	261.28	O=C1Nc2ccccc2/C/1=C/c3ccc4[nH]ncc4c3	4.3010	4.3391
21^	CHEMBL2407911	261.28	O=C1Nc2ccccc2/C/1=C\c3ccc4cn[nH]c4c3	4.3802	4.3380
22	CHEMBL2407909	262.27	O=C1Nc2ccccc2/C/1=C\c3ccc4nn[nH]c4c3	4.4624	4.3307
23	CHEMBL492008	264.22	NC(=O)c1sc2c(cc(cc2[n+]1[O-])C#N)[N+](=O)[O-]	3.9031	3.4373
24	CHEMBL1288582	264.28	COc1ccc2cc([nH]c2c1)c3n[nH]c4cccnc34	4.4771	4.3655
25	CHEMBL1689162	273.31	COc1cc(sc1C(=O)N)n2cnc3ccccc23	3.7251	1.4366
26^	CHEMBL407532	273.72	Nc1ncnc2NCCC(=Nc12)c3cccc(Cl)c3	5.0000	4.3362
27^	CHEMBL407265	273.72	Nc1ncnc2NCCC(=Nc12)c3ccc(Cl)cc3	5.0000	4.3307
28	CHEMBL454440	274.23	Cc1c(O)c(O)cc2Oc3cc(O)cc(O)c3C(=O)c12	3.9445	2.9942
29	CHEMBL2407912	279.27	Fc1ccc2NC(=O)\C(=C\c3ccc4cn[nH]c4c3)\c2c1	4.1761	4.2624
30*	CHEMBL2407913	279.27	Fc1ccc2NC(=O)\C(=C/c3ccc4cn[nH]c4c3)\c2c1	4.1761	4.2624
31	CHEMBL1270230	279.3	NC(=O)c1cc([nH]c1c2ccccc2)c3ccnc(N)n3	4.0000	4.1324
32	CHEMBL1957190	279.33	CC(C)n1c2CCCC(=O)c2c3C(=O)c4ccccc4c13	4.5441	3.8912

33	CHEMBL599248	280.28	COc1ccc2c(NC(=O)Cc3cnc4ccnn4c23)c1	5.0000	4.3318
34^	CHEMBL597005	281.27	COc1ccc2c(NC(=O)Cc3cnc4ncnn4c23)c1	5.0000	4.3223
35	CHEMBL3394760	288.34	OC(CN1CCCC1)Cn2cc(C(=O)O)c3ccccc23	3.6848	3.3405
36^	CHEMBL260620	288.74	Nc1nc(N)c2N=C(CCNc2n1)c3ccc(Cl)cc3	5.0000	4.3018
37	CHEMBL259381	288.74	Nc1nc(N)c2N=C(CCNc2n1)c3cccc(Cl)c3	5.0000	4.3072
38	CHEMBL3394758	290.36	CCN(CC)CC(O)Cn1cc(C(=O)O)c2ccccc12	3.2810	3.3359
39^	CHEMBL2407916	291.3	COc1ccc2NC(=O)\C(=C\c3ccc4cn[nH]c4c3)\c2c1	4.0000	4.3173
40	CHEMBL597207	294.26	O=C1Cc2cc3C(=O)NC(=O)Nc3nc2c4ccccc4N1	5.0000	4.0819
41^	CHEMBL610512	295.25	O=C1Cc2cc3C(=O)NC(=O)Nc3nc2c4ncccc4N1	5.0000	4.0724
42^	CHEMBL606119	295.25	O=C1Cc2cc3C(=O)NC(=O)Nc3nc2c4cnccc4N1	5.0000	4.0747
43	CHEMBL2407914	295.72	Clc1ccc2NC(=O)\C(=C\c3ccc4cn[nH]c4c3)\c2c1	4.0000	4.3483
44*	CHEMBL2407915	295.72	Clc1ccc2\C(=C/c3ccc4cn[nH]c4c3)\C(=O)Nc2c1	4.4150	4.3483
45^	CHEMBL442957	298.36	Cc1nc(C)c(s1)c2ccnc(Nc3ccc(O)cc3)n2	3.6990	4.3655
46	CHEMBL431336	299.35	Cc1nc(N)sc1c2ccnc(Nc3ccc(O)cc3)n2	4.3979	4.3655
47^	CHEMBL2036725	301.35	Cn1c(nc2ccccc12)c3ccnc(Nc4ccccc4)n3	4.7782	4.3655
48	CHEMBL6246	302.19	Oc1cc2C(=O)Oc3c(O)c(O)cc4C(=O)Oc(c1O)c2c34	3.5237	2.8122
49	CHEMBL50	302.24	OC1=C(Oc2cc(O)cc(O)c2C1=O)c3ccc(O)c(O)c3	3.7597	3.9529
50	CHEMBL603255	302.33	O=C1Cc2cnc(Nc3ccccc3)nc2c4ccccc4N1	5.0000	4.3413
51*	CHEMBL3394764	302.37	OC(CN1CCCC1)Cn2cc(CC(=O)O)c3ccccc23	2.1139	3.3971
52*	CHEMBL3394759	302.37	OC(CN1CCCCC1)Cn2cc(C(=O)O)c3ccccc23	3.3222	3.3412
53^	CHEMBL3394762	304.38	CCN(CC)CC(O)Cn1cc(CC(=O)O)c2ccccc12	3.4216	3.3933
54^	CHEMBL1088633	306.19	COc1cc2CCN=C(c3cccc(Cl)c3)c2cc1Cl	4.0000	4.4098
55	CHEMBL1094401	306.32	NC(=O)c1n[nH]c2c1CCc3cnc(Nc4ccccc4)nc23	2.3945	3.0427
56	CHEMBL476124	307.21	NC(=O)c1sc2c(cc(cc2[n+]1[O-])C(F)(F)F)[N+](=O)[O-]	2.6990	3.3427
57	CHEMBL3260250	311.33	CC(C)C1=CC(=O)C(=CC1=O)\C=N\OC(=O)c2ccccc2C	3.3636	3.6073
58*	CHEMBL47590	313.38	CNc1nc(C)c(s1)c2ccnc(Nc3ccc(O)cc3)n2	4.0000	4.3655
59	CHEMBL452932	315.37	C[C@H](Nc1cc2c(noc2cn1)c3ccccc3)c4ccccc4	3.2084	3.2607
60^	CHEMBL2036726	315.37	Cc1ccc(Nc2nccc(n2)c3nc4ccccc4n3C)cc1	5.0000	4.3655
61^	CHEMBL605324	316.36	Cc1ccc(Nc2ncc3CC(=O)Nc4ccccc4c3n2)cc1	5.0000	4.3415
62	CHEMBL3394763	316.39	OC(CN1CCCCC1)Cn2cc(CC(=O)O)c3ccccc23	2.6128	3.3977
63	CHEMBL3394768	316.39	OC(CN1CCCC1)Cn2cc(CCC(=O)O)c3ccccc23	3.4624	3.4283
64*	CHEMBL2036870	317.34	Cn1c(nc2ccccc12)c3ccnc(Nc4cccc(O)c4)n3	3.5682	4.3655
65	CHEMBL2036869	317.34	Cn1c(nc2ccccc12)c3ccnc(Nc4ccc(O)cc4)n3	4.0792	4.3655
66	CHEMBL3394761	317.38	CN1CCN(CC(O)Cn2cc(C(=O)O)c3ccccc23)CC1	3.5403	3.3263
67	CHEMBL2407918	318.33	CC(=O)Nc1ccc2NC(=O)\C(=C\c3ccc4cn[nH]c4c3)\c2c1	4.0000	4.2922
68^	CHEMBL2407919	318.33	CC(=O)Nc1ccc2\C(=C/c3ccc4cn[nH]c4c3)\C(=O)Nc2c1	4.0000	4.2922
69^	CHEMBL603545	318.33	Oc1ccccc1Nc2ncc3CC(=O)Nc4ccccc4c3n2	4.1461	2.8846

70	CHEMBL604545	318.33	Oc1cccc(Nc2ncc3CC(=O)Nc4ccccc4c3n2)c1	4.2788	4.3262
71	CHEMBL2407917	318.33	CNC(=O)c1ccc2NC(=O)\C(=C\c3ccc4cn[nH]c4c3)\c2c1	4.6902	4.2796
72^	CHEMBL605841	318.33	Oc1ccc(Nc2ncc3CC(=O)Nc4ccccc4c3n2)cc1	5.0000	4.3285
73	CHEMBL3394766	318.37	OC(CN1CCOCC1)Cn2cc(CC(=O)O)c3ccccc23	2.9823	3.3675
74^	CHEMBL602043	319.32	Oc1cccc(Nc2ncc3CC(=O)Nc4ccncc4c3n2)c1	3.3617	4.3132
75	CHEMBL597597	319.32	Oc1ccc(Nc2ncc3CC(=O)Nc4ccncc4c3n2)cc1	3.4150	4.3156
76	CHEMBL598633	319.32	Oc1ccc(Nc2ncc3CC(=O)Nc4cccnc4c3n2)cc1	4.7404	4.3100
77	CHEMBL606185	319.32	Oc1cccc(Nc2ncc3CC(=O)Nc4cccnc4c3n2)c1	5.0000	4.3076
78	CHEMBL559845	320.35	Cn1nc(C(=O)N)c2CCc3cnc(Nc4ccccc4)nc3c12	1.8325	3.0510
79^	CHEMBL1802870	320.35	CCc1nc(C)c(\C=C\2/C(=O)Nc3ccc(NC(=O)C#C)cc23)[nH]1	4.3010	3.6696
80*	CHEMBL1098060	321.33	Cn1nc(C(=O)O)c2CCc3cnc(Nc4ccccc4)nc3c12	2.0414	2.8015
81	CHEMBL2036871	321.33	CN1C(=O)N(C)c2nc(ccc2C1=O)c3nc4ccccc4n3C	5.0000	4.1792
82*	CHEMBL490992	321.4	C[C@H](Nc1cc2c(noc2cn1)c3cccs3)c4ccccc4	3.4781	3.2682
83	CHEMBL603481	322.32	CN1C(=O)N(C)c2nc3c(CC(=O)Nc4ccccc34)cc2C1=O	5.0000	4.1637
84	CHEMBL599055	323.31	CN1C(=O)N(C)c2nc3c(CC(=O)Nc4ccncc34)cc2C1=O	5.0000	4.1564
85	CHEMBL599056	323.31	CN1C(=O)N(C)c2nc3c(CC(=O)Nc4cccnc34)cc2C1=O	5.0000	4.1542
86^	CHEMBL599666	324.29	COc1ccc2c(NC(=O)Cc3cc4C(=O)NC(=O)Nc4nc23)c1	5.0000	4.0740
87	CHEMBL3260249	324.33	O=C(NNC(=O)c1ccc2OCCOc2c1)\C=C\c3ccccc3	1.9542	2.4275
88	CHEMBL599247	326.35	O=C1Cc2cnc3cc(nn3c2c4ccccc4N1)c5ccccc5	5.0000	4.3474
89	CHEMBL296586	327.18	Brc1ccc2[nH]c3c(CC(=O)Nc4ccccc34)c2c1	5.0000	4.3932
90^	CHEMBL598435	327.34	O=C1Cc2cnc3cc(nn3c2c4ncccc4N1)c5ccccc5	5.0000	4.3288
91^	CHEMBL598434	327.34	O=C1Cc2cnc3cc(nn3c2c4cnccc4N1)c5ccccc5	5.0000	4.3345
92	CHEMBL525907	328.41	C[C@H](Nc1cc2c(cn1)c(C)nn2c3ccccc3)c4ccccc4	3.1143	3.8049
93	CHEMBL489992	329.4	C[C@H](Nc1cc2c(noc2cn1)c3cccc(C)c3)c4ccccc4	2.7396	3.2607
94*	CHEMBL521761	329.4	C[C@H](Nc1cc2c(noc2cn1)c3ccc(C)cc3)c4ccccc4	3.0682	3.2607
95	CHEMBL491022	329.4	C[C@H](Nc1cc2c(Cc3ccccc3)noc2cn1)c4ccccc4	4.3010	3.2645
96^	CHEMBL3394767	330.42	OC(CN1CCCCC1)Cn2cc(CCC(=O)O)c3ccccc23	3.7566	3.4287
97*	CHEMBL2036868	331.37	COc1ccc(Nc2nccc(n2)c3nc4ccccc4n3C)cc1	4.1761	4.3655
98*	CHEMBL3394765	331.41	CN1CCN(CC(O)Cn2cc(CC(=O)O)c3ccccc23)CC1	3.2742	3.3833
99^	CHEMBL2407908	332.15	Oc1ccc(\C=C\2/C(=O)Nc3ccc(Br)cc23)cc1O	4.0000	4.2994
100*	CHEMBL598201	332.36	Cc1cc(O)ccc1Nc2ncc3CC(=O)Nc4ccccc4c3n2	4.3010	4.3289
101*	CHEMBL598025	332.36	COc1ccc(Nc2ncc3CC(=O)Nc4ccccc4c3n2)cc1	4.6721	4.3357
102	CHEMBL1277019	332.42	CCc1ccsc1c2cnc(N)c(n2)N3CCC(CC3)C(=O)O	3.9294	3.5029
103*	CHEMBL596988	333.34	COc1ccc(Nc2ncc3CC(=O)Nc4ccncc4c3n2)cc1	3.3979	4.3228
104	CHEMBL606255	333.34	COc1ccc(Nc2ncc3CC(=O)Nc4cccnc4c3n2)cc1	5.0000	4.3171
105	CHEMBL3309995	334.33	Nc1ccc2c(c1)nc(Cc3ccc4OCOc4c3)n5nc(N)nc25	4.0000	4.3655
106*	CHEMBL1094477	334.38	Cc1ccccc1Nc2ncc3CCc4c(nn(C)c4c3n2)C(=O)N	1.1761	3.0513

107	CHEMBL562104	334.38	CNC(=O)c1nn(C)c2c1CCc3cnc(Nc4ccccc4)nc23	3.6248	3.2081
108*	CHEMBL1097022	335.36	Cn1nc(C(=O)N)c2CCc3cnc(Nc4ccccc4N)nc3c12	2.1761	3.0393
109^	CHEMBL523634	335.42	C[C@H](Nc1cc2c(noc2cn1)c3cscc3C)c4ccccc4	2.8915	3.2671
110*	CHEMBL491193	335.44	C[C@H](Nc1cc2c(noc2cn1)C3CCCCC3C)c4ccccc4	3.7550	3.2791
111^	CHEMBL2036727	335.79	Cn1c(nc2ccccc12)c3ccnc(Nc4ccc(Cl)cc4)n3	5.0000	4.3655
112*	CHEMBL599844	336.78	Clc1ccccc1Nc2ncc3CC(=O)Nc4ccccc4c3n2	4.8633	4.3447
113	CHEMBL596807	336.78	Clc1ccc(Nc2ncc3CC(=O)Nc4ccccc4c3n2)cc1	5.0000	4.3436
114^	CHEMBL3309990	337.31	Nc1nc2c3c(F)cccc3nc(Cc4ccc5OCOc5c4)n2n1	4.0000	4.3655
115*	CHEMBL1095771	338.34	Cn1nc(C(=O)N)c2CCc3cnc(Nc4ccccc4F)nc3c12	2.0969	3.0154
116*	CHEMBL3260246	338.36	Cc1ccc(\C=C\C(=O)NNC(=O)c2ccc3OCCOc3c2)cc1	3.0212	2.4277
117	CHEMBL1222044	339.27	NC(=O)c1sc2c(cc(SC(F)(F)F)cc2[n+]1[O-])[N+](=O)[O-]	1.3010	3.3804
118	CHEMBL1087421	340.63	COc1cc2CCN=C(c3ccc(Cl)c(Cl)c3)c2cc1Cl	4.0000	4.4205
119	CHEMBL3260243	342.32	Fc1ccc(\C=C\C(=O)NNC(=O)c2ccc3OCCOc3c2)cc1	2.7709	2.3845
120	CHEMBL1956071	342.36	CCn1c(nc2cncc(C(=O)N3CC[C@H](N)C3)c12)c4nonc4N	4.0000	3.7377
121	CHEMBL489993	343.42	C[C@H](Nc1cc2c(noc2cn1)c3cc(C)cc(C)c3)c4ccccc4	2.5185	3.2607
122	CHEMBL2036867	345.4	CCOc1ccc(Nc2nccc(n2)c3nc4ccccc4n3C)cc1	5.0000	3.8589
123	CHEMBL3298400	345.46	CC(C)(C)CNC(=O)CC1CNC(=O)c2cc(cn12)c3ccsc3	4.0000	4.0868
124^	CHEMBL606410	346.38	CCOc1ccc(Nc2ncc3CC(=O)Nc4ccccc4c3n2)cc1	5.0000	3.8278
125^	CHEMBL596808	347.33	[O-][N+](=O)c1ccc(Nc2ncc3CC(=O)Nc4ccccc4c3n2)cc1	5.0000	4.3121
126	CHEMBL2036866	347.37	COc1ccc(Nc2nccc(n2)c3nc4ccccc4n3C)cc1O	3.0792	4.3655
127	CHEMBL605118	347.37	CCOc1ccc(Nc2ncc3CC(=O)Nc4ccncc4c3n2)cc1	3.2304	3.8124
128^	CHEMBL598837	347.37	CCOc1ccc(Nc2ncc3CC(=O)Nc4cccnc4c3n2)cc1	5.0000	3.8063
129	CHEMBL598418	348.36	COc1ccc2c(NC(=O)Cc3cnc(Nc4ccc(O)cc4)nc23)c1	2.8751	4.3079
130*	CHEMBL604912	348.36	COc1ccc2c(NC(=O)Cc3cnc(Nc4cccc(O)c4)nc23)c1	2.9243	4.3055
131	CHEMBL603463	348.36	COc1ccc(Nc2ncc3CC(=O)Nc4ccccc4c3n2)cc1O	3.7559	4.3206
132	CHEMBL1094735	348.4	CC(C)n1nc(C(=O)N)c2CCc3cnc(Nc4ccccc4)nc3c12	2.6335	3.0522
133*	CHEMBL1095457	349.39	CNc1ccccc1Nc2ncc3CCc4c(nn(C)c4c3n2)C(=O)N	2.0414	3.0440
134*	CHEMBL1098059	349.39	CCOC(=O)c1nn(C)c2c1CCc3cnc(Nc4ccccc4)nc23	4.0000	3.0684
135^	CHEMBL523286	349.81	C[C@H](Nc1cc2c(noc2cn1)c3cccc(Cl)c3)c4ccccc4	2.7959	3.2609
136*	CHEMBL1094135	350.37	COc1ccccc1Nc2ncc3CCc4c(nn(C)c4c3n2)C(=O)N	1.6232	1.5975
137^	CHEMBL1095448	350.37	COc1cccc(Nc2ncc3CCc4c(nn(C)c4c3n2)C(=O)N)c1	2.1303	3.0384
138^	CHEMBL1094322	350.37	COc1ccc(Nc2ncc3CCc4c(nn(C)c4c3n2)C(=O)N)cc1	2.4082	3.0403
139*	CHEMBL2036865	351.79	Cn1c(nc2ccccc12)c3ccnc(Nc4ccc(O)c(Cl)c4)n3	3.7782	4.3655
140	CHEMBL596598	352.34	COc1ccc2c(NC(=O)Cc3cc4C(=O)N(C)C(=O)N(C)c4nc23)c1	5.0000	4.1558
141*	CHEMBL598000	352.77	Oc1ccc(Nc2ncc3CC(=O)Nc4ccccc4c3n2)cc1Cl	3.9685	4.3313
142	CHEMBL599446	353.76	Oc1ccc(Nc2ncc3CC(=O)Nc4ccncc4c3n2)cc1Cl	3.1461	4.3184
143^	CHEMBL598431	353.76	Oc1ccc(Nc2ncc3CC(=O)Nc4cccnc4c3n2)cc1Cl	5.0000	4.3127

144	CHEMBL3260241	354.36	COc1cccc(\C=C\C(=O)NNC(=O)c2ccc3OCCOc3c2)c1	1.4771	2.4130
145^	CHEMBL3260247	354.36	COc1ccc(\C=C\C(=O)NNC(=O)c2ccc3OCCOc3c2)cc1	2.2788	2.4169
146^	CHEMBL603259	356.38	COc1ccc2c(NC(=O)Cc3cnc4cc(nn4c23)c5ccccc5)c1	4.2304	4.3267
147^	CHEMBL3104855	358.3	Cc1c(O)c(O)c2oc(cc2c1C=O)c3c(C)c(O)c(O)c(O)c3C=O	3.8000	3.2496
148*	CHEMBL3260244	358.78	Clc1ccc(\C=C\C(=O)NNC(=O)c2ccc3OCCOc3c2)cc1	3.1903	2.4303
149*	CHEMBL522618	359.38	C[C@H](Nc1cc2c(noc2cn1)c3ccc4OCOc4c3)c5ccccc5	2.3304	3.2452
150^	CHEMBL559639	360.41	Cn1nc(C(=O)NC2CC2)c3CCc4cnc(Nc5ccccc5)nc4c13	4.0000	3.2522
151	CHEMBL1230607	361.48	CC(C)CC(=O)Nc1n[nH]c2c1CN(C(=O)C3CCN(C)CC3)C2(C)C	4.0000	4.7381
152^	CHEMBL606395	362.38	COc1ccc(Nc2ncc3CC(=O)Nc4cc(OC)ccc4c3n2)cc1	2.5441	4.3151
153	CHEMBL1099105	362.39	CC(=O)c1cccc(Nc2ncc3CCc4c(nn(C)c4c3n2)C(=O)N)c1	2.0000	3.0275
154*	CHEMBL1099106	362.39	CC(=O)c1ccc(Nc2ncc3CCc4c(nn(C)c4c3n2)C(=O)N)cc1	2.2945	3.0308
155*	CHEMBL1099104	362.39	CC(=O)c1ccccc1Nc2ncc3CCc4c(nn(C)c4c3n2)C(=O)N	2.5391	3.0233
156	CHEMBL1095772	362.43	CC(C)c1ccccc1Nc2ncc3CCc4c(nn(C)c4c3n2)C(=O)N	2.5623	3.0517
157*	CHEMBL478028	362.86	C[C@H](Nc1cc2c(cn1)c(C)nn2c3cccc(Cl)c3)c4ccccc4	2.0828	3.8051
158	CHEMBL1945559	363.24	Cn1c2C(=O)NC3(CCNCC3)C(C#N)c2c4ccc(Cl)c(Cl)c14	4.0000	4.0312
159	CHEMBL1094815	363.37	Cn1nc(C(=O)N)c2CCc3cnc(Nc4ccccc4C(=O)N)nc3c12	3.3172	3.0133
160	CHEMBL3309997	363.37	OCCCNc1ccc2c(c1)nc(c3ccc4OCOc4c3)n5ncnc25	4.0000	3.8705
161*	CHEMBL2407896	363.41	O=C1Nc2ccccc2/C/1=C\c3ccc4c(\C=C\c5ccccc5)n[nH]c4c3	4.0000	4.3321
162^	CHEMBL2407746	364.4	O=C1Nc2ccccc2/C/1=C\c3ccc4c(\C=C\c5cccnc5)n[nH]c4c3	4.0000	4.3294
163*	CHEMBL2407745	364.4	O=C1Nc2ccccc2/C/1=C\c3ccc4c(\C=C\c5ccccn5)n[nH]c4c3	4.0000	4.3289
164	CHEMBL2407749	364.4	O=C1Nc2ccccc2/C/1=C\c3ccc4c(\C=C\c5ccncc5)n[nH]c4c3	4.0000	4.3298
165*	CHEMBL1094810	365.35	Cn1nc(C(=O)N)c2CCc3cnc(Nc4ccccc4[N+](=O)[O-])nc3c12	2.6884	2.9949
166^	CHEMBL1095132	365.35	Cn1nc(C(=O)N)c2CCc3cnc(Nc4ccc(cc4)[N+](=O)[O-])nc3c12	4.0000	3.0103
167	CHEMBL1094811	365.35	Cn1nc(C(=O)N)c2CCc3cnc(Nc4cccc(c4)[N+](=O)[O-])nc3c12	4.0000	3.0035
168	CHEMBL3741589	366.24	OC(=O)C1CN(Cc2ccc(OCc3ccc(Cl)c(Cl)c3)cc2)C1	4.0000	3.5104
169	CHEMBL261849	366.41	COc1ccc(COc2ccc(Cc3cnc(N)nc3N)cc2OC)cc1	4.3802	4.3655
170*	CHEMBL1094478	366.44	CSc1ccccc1Nc2ncc3CCc4c(nn(C)c4c3n2)C(=O)N	1.9868	3.0566
171	CHEMBL3344193	368.86	COc1cc(CNCc2ccncc2)ccc1OCc3ccc(Cl)cc3	0.4771	4.3655
172	CHEMBL3260248	369.33	[O-][N+](=O)c1ccc(\C=C\C(=O)NNC(=O)c2ccc3OCCOc3c2)cc1	2.0792	2.3628
173	CHEMBL3260236	369.33	[O-][N+](=O)c1ccccc1\C=C\C(=O)NNC(=O)c2ccc3OCCOc3c2	2.2788	2.3214
174^	CHEMBL3260242	369.33	[O-][N+](=O)c1cccc(\C=C\C(=O)NNC(=O)c2ccc3OCCOc3c2)c1	3.1903	2.3458
175	CHEMBL599224	369.42	CC(=O)N1CCC(CC1)Nc2ncc3CCc4c(nn(C)c4c3n2)C(=O)N	4.0000	3.0513
176^	CHEMBL3109404	372.5	COC(=O)c1cc(O)c(O)c(C[C@]2(C)[C@@H](C)CC[C@]3(C)[C@H]2CCC=C3C)c1	4.3365	3.4230
177	CHEMBL3104851	374.3	Cc1c(O)c(O)c2O[C@H]3O[C@H](C(=O)c4c(O)c(O)c(O)c(C)c34)c2c1C=O	3.5024	2.8791
178	CHEMBL491621	375.42	COc1ccc(cc1OC)c2noc3cnc(N[C@@H](C)c4ccccc4)cc23	3.6926	3.2452
179	CHEMBL604712	376.4	CC(C)(C)C(=O)N1Cc2c(NC(=O)c3cc(F)cc(F)c3)n[nH]c2C1(C)C	4.0000	4.4876
180*	CHEMBL597191	376.41	CCOc1ccc(Nc2ncc3CC(=O)Nc4cc(OC)ccc4c3n2)cc1	3.8633	3.8034

181*	CHEMBL2401965	376.45	CC[C@H]1N(C(C)C)c2nc(ncc2N(C)C1=O)n3ccnc3c4ccccc4	2.9395	3.7446
182^	CHEMBL3358966	377.39	COc1cc(cc(OC)c1OC)C2=NC(=CNC2=O)c3c[nH]c4ccccc34	5.0000	4.2226
183*	CHEMBL1097373	377.4	CC(=O)Nc1ccccc1Nc2ncc3CCc4c(nn(C)c4c3n2)C(=O)N	3.4019	3.0203
184*	CHEMBL597393	378.38	COc1ccc2c(NC(=O)Cc3cnc(Nc4ccc(OC)c(O)c4)nc23)c1	2.1461	4.3000
185*	CHEMBL1094484	378.38	COC(=O)c1ccccc1Nc2ncc3CCc4c(nn(C)c4c3n2)C(=O)N	3.0481	3.0105
186	CHEMBL3309998	378.38	Nc1nc2c3ccc(NCCO)cc3nc(Cc4ccc5OCOc5c4)n2n1	4.0000	3.8236
187	CHEMBL2407893	378.43	Cc1ccc2NC(=O)\C(=C\c3ccc4c(\C=C\c5ccncc5)n[nH]c4c3)\c2c1	4.0000	4.3317
188^	CHEMBL3353340	378.43	O=C1Nc2ccccc2[C@]13C[C@H]3c4ccc5c(\C=C\c6ccncc6)n[nH]c5c4	5.0000	4.7395
189	CHEMBL3814269	380.22	COc1ccc(Br)cc1\C=C\2/SC3=NC(=O)C(=NN3C2=O)C	3.5051	4.2110
190*	CHEMBL2407895	380.4	Oc1ccc2NC(=O)\C(=C\c3ccc4c(\C=C\c5ccncc5)n[nH]c4c3)\c2c1	4.0000	4.2800
191	CHEMBL2407903	380.62	COc1cc(\C=C\2/C(=O)Nc3ccc(Br)cc23)cc(Cl)c1O	3.5563	4.3280
192	CHEMBL597586	381.23	Brc1ccc(Nc2ncc3CC(=O)Nc4ccccc4c3n2)cc1	5.0000	4.3445
193^	CHEMBL599027	381.23	Brc1ccccc1Nc2ncc3CC(=O)Nc4ccccc4c3n2	5.0000	4.3460
194^	CHEMBL1094400	382.42	NC(=O)c1nn(c2ccccc2)c3c1CCc4cnc(Nc5ccccc5)nc34	4.0000	3.0074
195*	CHEMBL597596	382.8	COc1ccc2c(NC(=O)Cc3cnc(Nc4ccc(O)c(Cl)c4)nc23)c1	2.5563	4.3107
196^	CHEMBL2401964	383.47	CC[C@H]1N(C(C)C)c2nc(ncc2N(C)C1=O)c3cn[nH]c3c4nccs4	2.8865	3.7508
197	CHEMBL3109402	383.52	COc1cc2nc(C)oc2c(C[C@]3(C)[C@@H](C)CC[C@]4(C)[C@H]3CCC=C4C)c1O	5.0000	3.8106
198	CHEMBL3814827	386.41	FC(F)(F)c1ccc(OC(CCNCc2ccccn2)c3ccccc3)cc1	1.6990	3.6534
199^	CHEMBL2036728	386.45	Cn1c(nc2ccccc12)c3ccnc(Nc4ccc(cc4)N5CCOCC5)n3	5.0000	3.8472
200	CHEMBL1689161	386.47	COc1cc(sc1C(=O)N)n2cnc3ccc(OC4CCN(C)CC4)cc23	3.1239	0.8838
201	CHEMBL1289926	386.47	CNC(=O)c1ccccc1Sc2ccc3c(\C=C\c4ccccn4)n[nH]c3c2	4.0000	4.3581
202	CHEMBL1095709	388.35	NC(=O)c1nn(CC(F)(F)F)c2c1CCc3cnc(Nc4ccccc4)nc23	0.7782	2.4565
203^	CHEMBL1097424	388.35	Cn1nc(C(=O)N)c2CCc3cnc(Nc4cccc(c4)C(F)(F)F)nc3c12	1.7076	2.9747
204^	CHEMBL1097071	388.35	Cn1nc(C(=O)N)c2CCc3cnc(Nc4ccccc4C(F)(F)F)nc3c12	2.6355	2.9611
205	CHEMBL1097429	388.35	Cn1nc(C(=O)N)c2CCc3cnc(Nc4ccc(cc4)C(F)(F)F)nc3c12	2.9405	2.9854
206	CHEMBL1231522	388.42	COc1cc(cc(OC)c1OC)c2cnc(N)c(n2)N3CCC(CC3)C(=O)O	4.3541	3.4526
207	CHEMBL1094402	388.47	NC(=O)c1nn(C2CCCCC2)c3c1CCc4cnc(Nc5ccccc5)nc34	2.1553	3.0695
208*	CHEMBL563028	388.47	Cn1nc(C(=O)NC2CCCC2)c3CCc4cnc(Nc5ccccc5)nc4c13	4.0000	3.2584
209*	CHEMBL3394757	390.52	OC(CN1CCCCC1)Cn2cc(CCC(=O)c3ccccc3)c4ccccc24	3.2601	3.8710
210*	CHEMBL2407747	392.45	CCc1ccc2NC(=O)\C(=C\c3ccc4c(\C=C\c5cccnc5)n[nH]c4c3)\c2c1	4.0000	3.9347
211	CHEMBL553	393.44	COCCOc1cc2ncnc(Nc3cccc(c3)C#C)c2cc1OCCOC	5.0000	3.7011
212	CHEMBL522125	393.48	C[C@H](Nc1cc2c(noc2cn1)C3CCCN(CCC(=O)N)C3)c4ccccc4	3.8738	3.1445
213	CHEMBL188343	393.49	C1CCC(CC1)Nc2nc(Nc3ccc(cc3)N4CCOCC4)nc5[nH]cnc25	3.6990	3.8498
214	CHEMBL2407748	394.43	COc1ccc2NC(=O)\C(=C\c3ccc4c(\C=C\c5cccnc5)n[nH]c4c3)\c2c1	4.0000	4.3088
215*	CHEMBL2407894	394.43	COc1ccc2NC(=O)\C(=C\c3ccc4c(\C=C\c5ccncc5)n[nH]c4c3)\c2c1	4.0000	4.3091
216	CHEMBL2401961	394.45	CC(C)N1c2nc(ncc2N(C)C(=O)C1(C)C)n3ccnc3c4ccc(F)cc4	5.0000	4.7199
217^	CHEMBL2407904	394.65	COc1cc(\C=C\2/C(=O)N(C)c3ccc(Br)cc23)cc(Cl)c1O	4.0000	4.3689

218	CHEMBL3606021	395.42	NC(=O)c1cc(F)cc2CN(C3CCN(CC3)C4CCC(F)(F)CC4)C(=O)c12	4.0000	3.4451
219*	CHEMBL1094817	396.44	Cn1nc(C(=O)N)c2CCc3cnc(Nc4ccccc4c5ccccc5)nc3c12	3.1945	3.0379
220	CHEMBL563150	396.44	Cn1nc(C(=O)Nc2ccccc2)c3CCc4cnc(Nc5ccccc5)nc4c13	4.0000	3.1653
221	CHEMBL518060	396.61	CC(C)C\C=C\[C@@H](C)[C@H]1CC[C@H]2[C@H](CCc3cc(O)ccc3C)C(=O)CC[C@]12C	5.0000	5.0506
222	CHEMBL2324036	397.43	Cc1cc(c(OCCO)c(c1)c2ccc(cc2)c3nnn[nH]3)c4ccc(cc4)C#N	5.1614	3.7113
223	CHEMBL535	398.47	CCN(CC)CCNC(=O)c1c(C)[nH]c(\C=C\2/C(=O)Nc3ccc(F)cc23)c1C	4.0000	3.7220
224^	CHEMBL1922224	398.62	CC(C)C\C=C\[C@@H](C)[C@H]1CC[C@H]2[C@H](CCc3cc(O)ccc3C)[C@@H](O)CC[C@]12C	5.0000	4.8066
225^	CHEMBL456936	398.62	CC(C)CCC[C@@H](C)[C@H]1CC[C@H]2[C@H](CCc3cc(O)ccc3C)C(=O)CC[C@]12C	5.0000	5.0692
226*	CHEMBL2407750	398.84	Clc1ccc2NC(=O)\C(=C\c3ccc4c(\C=C\c5ccncc5)n[nH]c4c3)\c2c1	4.0000	4.3401
227	CHEMBL1094816	399.43	Cn1nc(C(=O)N)c2CCc3cnc(Nc4ccccc4S(=O)(=O)N)nc3c12	3.5721	2.9977
228	CHEMBL517956	400.64	CC(C)CCC[C@@H](C)[C@H]1CC[C@H]2[C@H](CCc3cc(O)ccc3C)[C@H](O)CC[C@]12C	5.0000	4.8143
229*	CHEMBL2401963	402.49	CC[C@H]1N(C2CCCC2)c3nc(ncc3N(C)C1=O)n4ccnc4c5ccccc5	0.9031	3.7675
230*	CHEMBL1098057	403.48	CN1CCC(CC1)n2nc(C(=O)N)c3CCc4cnc(Nc5ccccc5)nc4c23	4.0000	3.0373
231	CHEMBL1272089	404.35	Cn1nc(C(=O)N)c2CCc3cnc(Nc4ccccc4OC(F)(F)F)nc3c12	2.0682	1.5210
232	CHEMBL2401971	404.46	CC[C@H]1N([C@H]2CCOC2)c3nc(ncc3N(C)C1=O)n4ccnc4c5ccccc5	2.5798	3.7075
233^	CHEMBL2401972	404.46	CC[C@H]1N([C@@H]2CCOC2)c3nc(ncc3N(C)C1=O)n4ccnc4c5ccccc5	2.6812	3.7075
234^	CHEMBL2401960	406.46	CC(C)N1c2nc(ncc2N(C)C(=O)C13CCC3)n4ccnc4c5ccc(F)cc5	4.4031	4.7764
235	CHEMBL2407744	407.42	OC(=O)c1ccc(\C=C\c2n[nH]c3cc(\C=C/4\C(=O)Nc5ccccc45)ccc23)cc1	4.0000	3.9257
236*	CHEMBL2402085	407.45	CC[C@H]1N(c2cn[nH]c2)c3nc(ncc3N(C)C1=O)c4cn[nH]c4c5nccs5	3.4814	3.6202
237^	CHEMBL2047943	407.51	Cc1cc(ccc1Nc2nc(NC3CCCCC3)c4nc[nH]c4n2)N5CCOCC5	3.6990	3.8498
238*	CHEMBL490814	407.89	C[C@H](Nc1cc2c(noc2cn1)c3cc(Cl)cc(CCCO)c3)c4ccccc4	1.9956	3.2559
239*	CHEMBL2401970	408.43	CC[C@H]1N(C2COC2)c3nc(ncc3N(C)C1=O)n4ccnc4c5ccc(F)cc5	4.6990	3.6801
240^	CHEMBL1094488	410.47	Cn1nc(C(=O)N)c2CCc3cnc(Nc4ccccc4Cc5ccccc5)nc3c12	2.9745	3.0407
241*	CHEMBL2401974	410.47	CCC1N(c2ccccc2)c3nc(ncc3N(C)C1=O)n4ccnc4c5ccccc5	3.4518	3.6498
242	CHEMBL562030	410.47	Cn1nc(C(=O)NCc2ccccc2)c3CCc4cnc(Nc5ccccc5)nc4c13	4.0000	3.1913
243	CHEMBL1922225	410.63	CC(C)C(=C)CC[C@@H](C)[C@H]1CC[C@H]2[C@H](CCc3cc(O)ccc3C)C(=O)CC[C@]12C	5.0000	5.0561
244	CHEMBL1094820	411.46	Cn1nc(C(=O)N)c2CCc3cnc(Nc4ccccc4Nc5ccccc5)nc3c12	2.9773	3.0326
245	CHEMBL2402084	411.48	CC[C@H]1N([C@H]2CCOC2)c3nc(ncc3N(C)C1=O)c4cn[nH]c4c5nccs5	3.1492	3.7138
246*	CHEMBL1096095	412.44	Cn1nc(C(=O)N)c2CCc3cnc(Nc4ccccc4Oc5ccccc5)nc3c12	2.4440	1.5861
247	CHEMBL2402077	412.45	CC[C@H]1N(c2cncnc2)c3nc(ncc3N(C)C1=O)n4ccnc4c5ccccc5	3.8899	3.5898
248^	CHEMBL515982	412.65	CC(C)C(=C)CC[C@@H](C)[C@H]1CC[C@H]2[C@H](CCc3cc(O)ccc3C)[C@H](O)CC[C@]12C	5.0000	5.4551

249	CHEMBL3798011	413.47	C[C@@H](N1c2cc(ccc2C(=O)NC1(C)C)N3C(=O)Nc4ncccc34)c5ccccc5	3.0000	3.6268
250^	CHEMBL3109401	413.59	CC(C)CCNC1=CC(=O)C(=C(C[C@]2(C)[C@@H](C)CC[C@]3(C)[C@H]2CCC=C3C)C1=O)O	5.0000	4.5329
251	CHEMBL456294	414.62	CC(C)CCC[C@@H](C)[C@H]1[C@@H](O)C[C@H]2[C@H](CCc3cc(O)ccc3C)C(=O)CC[C@]12C	5.0000	4.9738
252^	CHEMBL2401957	415.45	CCC1N(c2cnn(C)c2)c3nc(ncc3N(C)C1=O)c4cn[nH]c4c5ccccn5	3.2601	3.6102
253^	CHEMBL1276848	416.47	COc1cc(cc(OC)c1OC)c2cnc(N)c(n2)N3CC[C@H]([C@@H](C)[C@H]3C)C(=O)O	4.2878	3.4501
254^	CHEMBL464351	416.64	CC(C)CCC[C@@H](C)[C@H]1[C@@H](O)C[C@H]2[C@H](CCc3cc(O)ccc3C)[C@H](O)CC[C@]12C	5.0000	4.7906
255	CHEMBL1098058	417.51	NC(=O)c1nn(CCN2CCCCC2)c3c1CCc4cnc(Nc5ccccc5)nc34	4.0000	3.0144
256*	CHEMBL2402079	418.43	CCC1N(c2cc[nH]n2)c3nc(ncc3N(C)C1=O)n4ccnc4c5ccc(F)cc5	3.5340	3.5734
257^	CHEMBL2402078	418.43	CC[C@H]1N(c2cn[nH]c2)c3nc(ncc3N(C)C1=O)n4ccnc4c5ccc(F)cc5	3.9380	3.5887
258^	CHEMBL445162	418.85	CS(=O)(=O)c1cccc(Nc2nccc(Nc3c(Cl)ccc4OCOc34)n2)c1	4.4771	2.9271
259*	CHEMBL1269042	419.36	Cn1nc(C(=O)N)c2CCc3cnc(Nc4cc(N)ccc4OC(F)(F)F)nc3c12	1.8633	1.5112
260	CHEMBL1290746	420.4	Cc1ccc(cc1NC(=O)c2cnn(c2N)c3ccccc3F)C(=O)Nc4ccon4	4.1761	4.1126
261	CHEMBL2407897	420.51	CN(C)Cc1ccc(\C=C\c2n[nH]c3cc(\C=C/4\C(=O)Nc5ccccc45)ccc23)cc1	4.0000	4.3316
262	CHEMBL491021	420.89	C[C@H](Nc1cc2c(noc2cn1)c3cc(Cl)cc(CCC(=O)N)c3)c4ccccc4	1.7076	3.1145
263	CHEMBL2402086	421.48	CCC1N(c2cnn(C)c2)c3nc(ncc3N(C)C1=O)c4cn[nH]c4c5nccs5	3.2601	3.6235
264	CHEMBL2401973	422.46	CC[C@H]1N([C@H]2CCOC2)c3nc(ncc3N(C)C1=O)n4ccnc4c5ccc(F)cc5	3.8768	3.6823
265*	CHEMBL1094821	424.45	Cn1nc(C(=O)N)c2CCc3cnc(Nc4ccccc4C(=O)c5ccccc5)nc3c12	3.2942	3.0119
266*	CHEMBL1808335	424.5	Cn1nc(C(=O)NCCc2ccccc2)c3CCc4cnc(Nc5ccccc5)nc4c13	4.0000	3.2068
267*	CHEMBL2402083	425.51	CC[C@H]1N(C2CCOCC2)c3nc(ncc3N(C)C1=O)c4cn[nH]c4c5nccs5	2.3979	3.7327
268	CHEMBL590109	425.51	NCCn1cc(c2cc(c3cc4ccccc4s3)c5[nH]ncc5c2)c6nc(N)ncc16	4.0000	3.8238
269*	CHEMBL493326	427.9	COc1ccc2ncn(c3cc(O[C@H](C)c4ccccc4Cl)c(s3)C(=O)N)c2c1	0.0000	0.7523
270	CHEMBL598024	428.23	Ic1ccc(Nc2ncc3CC(=O)Nc4ccccc4c3n2)cc1	4.1761	4.3446
271	CHEMBL428496	428.43	COC[C@H]1OC(=O)c2coc3C(=O)C4=C([C@@H](C[C@@]5(C)[C@H]4CCC5=O)OC(=O)C)[C@]1(C)c23	1.3802	3.0378
272	CHEMBL1094822	428.51	Cn1nc(C(=O)N)c2CCc3cnc(Nc4ccccc4Sc5ccccc5)nc3c12	3.3081	3.0453
273^	CHEMBL2380587	428.53	CC(=O)Nc1cccc(c1)c2n[nH]c3cc(c(NC4CCCCC4)cc23)c5cnn(C)c5	3.6990	3.8722
274*	CHEMBL2401966	430.43	CC[C@H]1N(CCC(F)(F)F)c2nc(ncc2N(C)C1=O)n3ccnc3c4ccccc4	2.3979	3.4325
275	CHEMBL1689163	431.43	CC(Oc1cc(sc1C(=O)N)n2cnc3ccccc23)c4ccccc4C(F)(F)F	0.9542	0.5073
276*	CHEMBL599428	431.49	Cn1nc(C(=O)N)c2CCc3cnc(NC4CCN(CC4)C(=O)c5ccccc5)nc3c12	4.0000	3.0428
277	CHEMBL3809829	431.53	COc1cc(ccc1Nc2ncc3ccnc(N[C@@H](C)C(C)(C)C)c3n2)c4cnn(C)c4	5.0000	3.3365
278	CHEMBL2402081	432.45	CC[C@H]1N(c2ccn(C)n2)c3nc(ncc3N(C)C1=O)n4ccnc4c5ccc(F)cc5	3.1303	3.5767
279*	CHEMBL2402080	432.45	CC[C@H]1N(c2cnn(C)c2)c3nc(ncc3N(C)C1=O)n4ccnc4c5ccc(F)cc5	3.5172	3.5920
280*	CHEMBL2402082	432.45	CC[C@H]1N(c2nc(ncc2N(C)C1=O)n3ccnc3c4ccc(F)cc4)c5ccnn5C	4.4594	3.5860
281^	CHEMBL3353347	434.53	CN(C)Cc1ccc(\C=C\c2n[nH]c3cc(ccc23)[C@@H]4C[C@@]45C(=O)Nc6ccccc56)cc1	5.0000	4.7421

282	CHEMBL3310151	435.45	CN1CCN(CC1)c2cc(F)c3c(c2)nc(Cc4ccc5OCOc5c4)n6nc(N)nc36	4.0000	3.8514
283	CHEMBL2407905	439.1	COc1cc(\C=C\2/C(=O)Nc3ccc(Br)cc23)cc(Br)c1OC	4.0000	4.3469
284*	CHEMBL1614712	439.55	Nc1ncnc2c1c(cn2[C@@H]3C[C@H](CN4CCC4)C3)c5cccc(OCc6ccccc6)c5	4.0000	3.8551
285	CHEMBL3651966	440.46	C[C@@H]1C[C@H](N)C[C@@H](C1)c2ccncc2NC(=O)c3ccc(F)c(n3)c4c(F)cccc4F	4.0000	3.0405
286	CHEMBL489083	441.5	NC(=N)SCCCN1C(=O)C2=C(C1=O)n3c(cc4ccccc34)c5cccc6ccn2c56	3.9956	3.6260
287^	CHEMBL2401969	442.44	CC[C@H]1N(C2CC(F)(F)C2)c3nc(ncc3N(C)C1=O)n4ccnc4c5ccc(F)cc5	3.9727	3.5521
288^	CHEMBL514162	443.9	COc1cc2ncn(c3cc(OCc4ccccc4Cl)c(s3)C(=O)N)c2cc1OC	0.3010	1.4208
289	CHEMBL604087	445.51	COc1ccc(cc1OC)C(=O)NCc2cccc(c2)C(=O)Nc3ccc4CCNCc4c3	4.0000	2.7152
290	CHEMBL2401975	446.45	CCC1N(c2ccc(F)cc2)c3nc(ncc3N(C)C1=O)n4ccnc4c5ccc(F)cc5	3.3692	3.5556
291	CHEMBL2401967	448.42	CC[C@H]1N(CCC(F)(F)F)c2nc(ncc2N(C)C1=O)n3ccnc3c4ccc(F)cc4	3.5502	3.4086
292^	CHEMBL1094408	448.52	COc1ccc(cc1Nc2ncc3CCc4c(nn(C)c4c3n2)C(=O)N)N5CCN(C)CC5	0.8451	1.5924
293*	CHEMBL1095717	448.52	COc1cc(ccc1Nc2ncc3CCc4c(nn(C)c4c3n2)C(=O)N)N5CCN(C)CC5	1.6021	1.5931
294	CHEMBL2332840	448.79	FC(F)(F)c1cc(NC(=O)Nc2ccc(Oc3ncnc4[nH]ncc34)cc2)ccc1Cl	4.0000	4.0561
295	CHEMBL2401956	450.44	CC[C@H]1N(c2cnn(C)c2)c3nc(ncc3N(C)C1=O)c4cn[nH]c4c5ccc(F)cc5F	3.1004	3.5593
296	CHEMBL2407898	450.53	COc1ccc2NC(=O)\C(=C\c3ccc4c(\C=C\c5ccc(CN(C)C)cc5)n[nH]c4c3)\c2c1	4.0000	4.3110
297	CHEMBL2380586	450.56	Cn1cc(cn1)c2cc3[nH]nc(c4cccc(c4)S(=O)(=O)N)c3cc2NC5CCCCC5	3.6990	3.9089
298	CHEMBL1241855	451.49	COc1cc(OC)c(\C=C\S(=O)(=O)Cc2ccc(OC)c(NCC(=O)O)c2)c(OC)c1	0.9542	2.4431
299	CHEMBL2443138	452.5	CN(C)c1ccc(Oc2cc(O)cc(O)c2c3onc(c3)C(=O)NC4CCN(C)CC4)cc1	1.0000	2.0277
300	CHEMBL497949	452.55	NC(=O)c1ccc2\C(=C(\Nc3ccc(CN4CCCCC4)cc3)/c5ccccc5)\C(=O)Nc2c1	4.0000	3.7482
301^	CHEMBL1808334	452.55	CCc1cccc(CC)c1NC(=O)c2nn(C)c3c2CCc4cnc(Nc5ccccc5)nc34	4.0000	5.8629
302*	CHEMBL2402076	453.47	CCC1N(c2ccc(cc2)C#N)c3nc(ncc3N(C)C1=O)n4ccnc4c5ccc(F)cc5	3.5752	3.5748
303^	CHEMBL2443044	454.43	CN1CCC(CC1)NC(=O)c2cc(on2)c3c(O)cc(O)cc3Oc4ccc(cc4)[N+](=O)[O-]	1.0000	1.9907
304	CHEMBL2333365	455.5	Cc1c(F)c(ccc1C(=O)N2CCOc3ccc(cc3C2)c4ccc(N)nc4)S(=O)(=O)C	4.0000	3.5339
305	CHEMBL2401968	456.46	CCC1N(C2CCC(F)(F)C2)c3nc(ncc3N(C)C1=O)n4ccnc4c5ccc(F)cc5	2.5051	3.5542
306	CHEMBL222419	457.93	COc1cc2ncn(c3cc(O[C@H](C)c4ccccc4Cl)c(s3)C(=O)N)c2cc1OC	-0.0969	0.7374
307	CHEMBL521806	457.93	COc1cc2ncn(c3cc(O[C@@H](C)c4ccccc4Cl)c(s3)C(=O)N)c2cc1OC	1.4771	0.7374
308	CHEMBL1089082	459.42	FC(F)(F)c1cccc(c1)C(=O)Nc2cccc(c2)c3ccnc4cc(nn34)c5ccncc5	4.6990	4.0695
309^	CHEMBL3218002	459.48	ONC(=O)\C=C\c1ccn(c1)S(=O)(=O)c2ccc3ncnc(Nc4cccc(c4)C#C)c3c2	5.0000	4.0324
310^	CHEMBL1095716	460.53	CN1CCN(CC1)c2ccc(C(=O)C)c(Nc3ncc4CCc5c(nn(C)c5c4n3)C(=O)N)c2	2.0374	3.0182
311	CHEMBL1096028	460.53	CN1CCN(CC1)c2ccc(Nc3ncc4CCc5c(nn(C)c5c4n3)C(=O)N)c(c2)C(=O)C	2.6665	3.0189
312*	CHEMBL1097374	460.53	CN1CCN(CC1)c2cccc(Nc3ncc4CCc5c(nn(C)c5c4n3)C(=O)N)c2C(=O)C	3.3120	3.0182
313	CHEMBL1933578	461.44	Cn1cc(cc1c2ccnc(Nc3cc(ccc3OC(F)(F)F)N4CCNCC4)n2)C(=O)N	1.2553	2.1318
314^	CHEMBL522709	461.46	COc1ccc2ncn(c3cc(O[C@H](C)c4ccccc4C(F)(F)F)c(s3)C(=O)N)c2c1	0.4771	0.4891
315^	CHEMBL2401959	462.44	CCC1(C)N(CCC(F)(F)F)c2nc(ncc2N(C)C1=O)n3ccnc3c4ccc(F)cc4	3.8549	4.2792
316	CHEMBL2407899	462.55	CN1CCN(CC1)c2ccc(\C=C\c3n[nH]c4cc(\C=C/5\C(=O)Nc6ccccc56)ccc34)cn2	4.0000	3.8629
317	CHEMBL1689159	462.56	CN1CCC(CC1)Oc2ccc3ncn(c4cc(OCc5ccccc5)c(s4)C(=O)N)c3c2	1.8388	0.8729
318^	CHEMBL1200485	464.82	CNC(=O)c1cc(Oc2ccc(NC(=O)Nc3ccc(Cl)c(c3)C(F)(F)F)cc2)ccn1	4.0000	2.3959

319*	CHEMBL2443139	466.53	CC(C)Nc1ccc(Oc2cc(O)cc(O)c2c3onc(c3)C(=O)NC4CCN(C)CC4)cc1	1.0000	2.0272
320	CHEMBL388978	466.53	CN[C@@H]1C[C@H]2O[C@@](C)([C@@H]1OC)n3c4ccccc4c5c6CNC(=O)c6c7c8cc ccc8n2c7c35	2.5441	3.5740
321	CHEMBL3745885	467.45	CN1C(=O)C(=Cc2cnc(Nc3ccc4[nH]ccc4c3)nc12)S(=O)(=O)c5ccc(F)cc5F	4.0000	3.9785
322	CHEMBL1242101	468.46	CC(=O)Nc1ccc(Nc2ncc(c(Nc3ccccc3C(=O)c4ccccc4)n2)[N+](=O)[O-])cc1	2.9542	4.2571
323	CHEMBL2178352	468.6	CN1CCN(CC1)c2ccc(Nc3ncc4nc(Nc5ccccc5)n(C6CCCC6)c4n3)cc2	4.0000	3.8179
324	CHEMBL475817	469.24	Nc1ncc(NC(=O)c2cc(NC(=O)c3cccc(c3)C(F)(F)F)ccc2Cl)cc1Cl	4.4771	4.0384
325	CHEMBL1209674	474.96	C[C@@H](Oc1cc(sc1C(=O)N)n2cnc3cc(ccc23)c4ccncc4)c5ccccc5Cl	0.0000	0.7535
326*	CHEMBL1209675	474.96	C[C@@H](Oc1cc(sc1C(=O)N)n2cnc3ccc(cc23)c4ccncc4)c5ccccc5Cl	0.3010	0.7505
327^	CHEMBL1933576	475.47	CN1CCN(CC1)c2ccc(OC(F)(F)F)c(Nc3nccc(n3)c4cc(cn4C)C(=O)N)c2	1.1761	2.1322
328*	CHEMBL1689160	476.59	CC(Oc1cc(sc1C(=O)N)n2cnc3ccc(OC4CCN(C)CC4)cc23)c5ccccc5	1.8195	0.7471
329	CHEMBL259084	476.86	OC(=O)c1ccc(Nc2ncc3CN=C(c4cc(Cl)ccc4c3n2)c5c(F)cccc5F)cc1	4.7243	3.8966
330	CHEMBL514499	477.46	COc1cc2ncn(c3cc(OCc4ccccc4C(F)(F)F)c(s3)C(=O)N)c2cc1OC	0.3010	1.3125
331	CHEMBL3330409	477.58	CCc1ccccc1C(N(C)C)C(=O)Nc2ccc3[nH]nc(c4cccc(c4)S(=O)(=O)N)c3c2	4.6990	3.8792
332*	CHEMBL1209842	477.97	C[C@@H](Oc1cc(sc1C(=O)N)n2cnc3cc(ccc23)c4cnn(C)c4)c5ccccc5Cl	0.0000	0.7525
333^	CHEMBL1209843	477.97	C[C@@H](Oc1cc(sc1C(=O)N)n2cnc3ccc(cc23)c4cnn(C)c4)c5ccccc5Cl	0.0000	0.7493
334^	CHEMBL1242977	479.45	CNc1nccc(n1)c2cccnc2Oc3ccc(NC(=O)c4cccc(c4)C(F)(F)F)cc3C	3.9031	4.0622
335^	CHEMBL3330410	481.59	NS(=O)(=O)c1cccc(c1)c2n[nH]c3ccc(NC(=O)C(N4CCCC4)c5ccsc5)cc23	4.6990	3.9151
336	CHEMBL1808336	482.58	CCc1cccc(CC)c1NC(=O)c2nn(C)c3c2CCc4cnc(Nc5ccccc5OC)nc34	4.0000	4.4094
337	CHEMBL525203	484.55	COC(=O)c1ccc2\C(=C(\Nc3ccc(CN(C)C(=O)N(C)C)cc3)/c4ccccc4)\C(=O)Nc2c1	4.0000	4.1477
338	CHEMBL3218000	485.54	Nc1ccccc1NC(=O)c2ccc(COc3ccc4ncnc(Nc5cccc(c5)C#C)c4c3)cc2	5.0000	4.2591
339^	CHEMBL1794067	487.96	C[C@@H](Oc1cc(sc1C(=O)N)n2cnc3ccc(OC[C@@H](O)CO)cc23)c4ccccc4Cl	0.0000	0.7211
340	CHEMBL1271644	488.47	Cn1nc(C(=O)N)c2CCc3cnc(Nc4cc(ccc4OC(F)(F)F)N5CCNCC5)nc3c12	0.3010	1.5155
341	CHEMBL1793891	488.47	CN1CCN(CC1)c2ccc(OC(F)(F)F)c(Nc3ncc4CCc5c(n[nH]c5c4n3)C(=O)N)c2	0.6021	1.5076
342^	CHEMBL1271591	488.47	Cn1nc(C(=O)N)c2CCc3cnc(Nc4cc(NC5CCNC5)ccc4OC(F)(F)F)nc3c12	1.8865	1.5142
343	CHEMBL1774332	488.47	Cn1nc(C(=O)N)c2CCc3cnc(Nc4cc(NC5CCCN5)ccc4OC(F)(F)F)nc3c12	1.9191	1.5088
344	CHEMBL1243200	488.54	CNc1nccc(n1)c2cccnc2Oc3ccc(NC(=O)c4ccccc4Nc5ccccc5)cc3	3.0000	4.2639
345^	CHEMBL2042135	490.54	C[C@@H](Oc1cc(ccc1C(=O)N)c2cc(cnc2N)c3cc(CN(C)C)cs3)\C=C/C(F)(F)F	3.7649	3.2318
346	CHEMBL2042041	490.54	CC(Oc1cc(ccc1C(=O)N)c2cc(cnc2N)c3cc(CN(C)C)cs3)\C=C/C(F)(F)F	3.9248	3.2318
347*	CHEMBL2042042	490.54	C[C@H](Oc1cc(ccc1C(=O)N)c2cc(cnc2N)c3cc(CN(C)C)cs3)\C=C/C(F)(F)F	4.6990	3.2318
348^	CHEMBL2407900	492.57	COc1ccc2NC(=O)\C(=C\c3ccc4c(\C=C\c5ccc(nc5)N6CCN(C)CC6)n[nH]c4c3)\c 2c1	4.0000	3.8399
349	CHEMBL1092830	494.45	Cc1oc(c(NC(=O)\N=C/2\NC=C(CCNc3cc(Nc4nc[nH]n4)ncn3)S2)c1)C(F)(F)F	2.8573	3.3796
350	CHEMBL3109933	494.97	CN(C)C(=O)c1ccc(Nc2cc3c(cn2)cc(c4cnn(C)c4)n3C(=O)OC(C)(C)C)c(Cl)c1	5.0000	4.5995
351*	CHEMBL1209758	496.5	C[C@@H](Oc1cc(sc1C(=O)N)n2cnc3cc(ccc23)c4ccc[nH]4)c5ccccc5C(F)(F)F	0.6990	0.4939
352*	CHEMBL1209759	497.49	C[C@@H](Oc1cc(sc1C(=O)N)n2cnc3cc(ccc23)c4cn[nH]c4)c5ccccc5C(F)(F)F	0.0000	0.4886
353	CHEMBL1964261	499.02	C[C@@H](Oc1cc(sc1C(=O)N)n2cnc3ccc(OCCCN(C)C)cc23)c4ccccc4Cl	0.6021	0.7511

354*	CHEMBL1774327	499.49	CN1CCC(=CC1)c2ccc(OC(F)(F)F)c(Nc3ncc4CCc5c(nn(C)c5c4n3)C(=O)N)c2	0.4771	1.5159
355*	CHEMBL1774328	500.52	CN1CCC(CC1)c2ccc(OC(F)(F)F)c(Nc3ncc4CCc5c(cn(C)c5c4n3)C(=O)N)c2	0.7782	2.1287
356*	CHEMBL1933582	501.5	CN1CCN(CC1)c2ccc(OC(F)(F)F)c(Nc3nccc(n3)c4cc5C(=O)NCCc5n4C)c2	0.9542	2.3318
357	CHEMBL2401958	502.39	CN1C(=O)C(CC(F)(F)F)N(CCC(F)(F)F)c2nc(ncc12)n3ccnc3c4ccc(F)cc4	3.5416	2.8772
358	CHEMBL1272140	502.49	CN1CCN(CC1)c2ccc(OC(F)(F)F)c(Nc3ncc4CCc5c(nn(C)c5c4n3)C(=O)N)c2	0.4771	1.5159
359*	CHEMBL1630117	504.9	ONC(=O)c1oc(cc1)c2ccc3ncnc(Nc4ccc(OCc5cccc(F)c5)c(Cl)c4)c3c2	3.3010	4.0279
360	CHEMBL1933579	505.49	CN1CCN(CC1)c2ccc(OC(F)(F)F)c(Nc3nccc(n3)c4cc(cn4CCO)C(=O)N)c2	2.1790	1.9956
361*	CHEMBL1209603	507.53	C[C@@H](Oc1cc(sc1C(=O)N)n2cnc3cc(ccc23)c4ccccc4)c5ccccc5C(F)(F)F	0.7782	0.4953
362*	CHEMBL1209604	507.53	C[C@@H](Oc1cc(sc1C(=O)N)n2cnc3ccc(cc23)c4ccccc4)c5ccccc5C(F)(F)F	1.2553	0.4931
363*	CHEMBL2392552	507.63	COc1cc(ccc1Nc2ncc3N(C)C(=O)CCN(C4CCCC4)c3n2)C(=O)NC5CCN(C)CC5	1.0792	1.2011
364	CHEMBL2348417	507.99	OCCNC(=O)C1=Cc2c(Nc3ccc(Oc4cccc5sccc45)c(Cl)c3)ncnc2NCC1	4.0000	3.7126
365	CHEMBL1209605	508.51	C[C@@H](Oc1cc(sc1C(=O)N)n2cnc3cc(ccc23)c4ccncc4)c5ccccc5C(F)(F)F	0.0000	0.4903
366^	CHEMBL1209676	508.51	C[C@@H](Oc1cc(sc1C(=O)N)n2cnc3cc(ccc23)c4cccnc4)c5ccccc5C(F)(F)F	0.0000	0.4895
367*	CHEMBL1209606	508.51	C[C@@H](Oc1cc(sc1C(=O)N)n2cnc3ccc(cc23)c4ccncc4)c5ccccc5C(F)(F)F	0.3010	0.4874
368	CHEMBL1209677	508.51	C[C@@H](Oc1cc(sc1C(=O)N)n2cnc3ccc(cc23)c4cccnc4)c5ccccc5C(F)(F)F	0.3010	0.4863
369*	CHEMBL1209756	508.51	C[C@@H](Oc1cc(sc1C(=O)N)n2cnc3cc(ccc23)c4ccccn4)c5ccccc5C(F)(F)F	0.7782	0.4885
370	CHEMBL3785951	508.59	CC(=O)N1CCN(CC1)[C@@H]2C[C@@H](C2)n3cc(c4cc(OC[C@@H]5CCCO5)ccc4 F)c6c(N)ncnc36	4.0000	3.7931
371	CHEMBL3623375	509.62	CN(C)CCCNC(=O)c1csc2nc(cn12)c3ccc(NC(=O)Nc4cc(on4)C(C)(C)C)cc3	4.0000	4.0254
372	CHEMBL1794070	511.04	C[C@@H](Oc1cc(sc1C(=O)N)n2cnc3ccc(OC4CCN(C)CC4)cc23)c5ccccc5Cl	0.3010	0.7539
373	CHEMBL1209841	511.52	C[C@@H](Oc1cc(sc1C(=O)N)n2cnc3cc(ccc23)c4cnn(C)c4)c5ccccc5C(F)(F)F	0.0000	0.4893
374^	CHEMBL3297762	512.09	CN1CCN(C[C@@H](NC(=O)C[C@H]2CNC(=O)c3cc(cn23)c4cccc(Cl)c4)C5CCCC C5)CC1	4.0000	3.1847
375	CHEMBL460702	512.89	CCOC1=C(C(=O)Nc2ccc(Oc3ccnc(N)c3Cl)c(F)c2)C(=O)N(C=C1)c4ccc(F)cc4	3.3010	3.3760
376	CHEMBL1209757	513.55	C[C@@H](Oc1cc(sc1C(=O)N)n2cnc3cc(ccc23)c4cccs4)c5ccccc5C(F)(F)F	0.4771	0.5048
377^	CHEMBL2392548	513.58	CN1CCC(CC1)NC(=O)c2ccc(Nc3ncc4N(C)C(=O)C(F)(F)CN(C5CCCC5)c4n3)cc2	0.0000	2.9471
378*	CHEMBL1793896	514.5	CN1CCN(CC1)c2ccc(OC(F)(F)F)c(Nc3ncc4CCc5c(nn(C=C)c5c4n3)C(=O)N)c2	0.7782	1.4843
379	CHEMBL1087397	515.53	CN(C)Cc1ccc(cc1)c2cnn3c(ccnc23)c4cccc(NC(=O)c5cccc(c5)C(F)(F)F)c4	3.3010	4.0717
380^	CHEMBL1243326	515.54	CNc1nccc(n1)c2cccnc2Oc3ccc(Nc4nnc(c5ccc(F)cc5)c6ccccc46)cc3	4.3979	4.3655
381	CHEMBL1242482	516.34	OC(=O)c1ccc(cc1)C(=O)\C(=C/c2ccc(F)c(c2)[N+](=O)[O-])\SCc3ccc(Br)cc3	4.6990	3.8629
382*	CHEMBL1774330	516.48	Cn1nc(C(=O)N)c2CCc3cnc(Nc4cc(NC(=O)[C@@H]5CCCN5)ccc4OC(F)(F)F)nc3c1 2	1.6435	1.4364
383	CHEMBL1793893	516.52	CCn1nc(C(=O)N)c2CCc3cnc(Nc4cc(ccc4OC(F)(F)F)N5CCN(C)CC5)nc3c12	0.7782	1.5183
384	CHEMBL1271590	516.52	CCN1CCN(CC1)c2ccc(OC(F)(F)F)c(Nc3ncc4CCc5c(nn(C)c5c4n3)C(=O)N)c2	0.9031	1.5163
385^	CHEMBL1272195	516.52	CN1CCCN(CC1)c2ccc(OC(F)(F)F)c(Nc3ncc4CCc5c(nn(C)c5c4n3)C(=O)N)c2	1.3979	1.5163
386	CHEMBL1272300	516.52	CN1CCC(CC1)Nc2ccc(OC(F)(F)F)c(Nc3ncc4CCc5c(nn(C)c5c4n3)C(=O)N)c2	2.3010	1.5154
387*	CHEMBL1271645	518.49	Cn1nc(C(=O)N)c2CCc3cnc(Nc4cc(ccc4OC(F)(F)F)N5CC[N+](C)([O-])CC5)nc3c12	2.1038	1.5036

388	CHEMBL72365	520.94	Oc1c(Br)cc(\C=C\2/C(=O)Nc3ccc(I)cc23)cc1Br	3.6628	4.3567
389^	CHEMBL1630118	520.96	ONC(=O)c1ccc(s1)c2ccc3ncnc(Nc4ccc(OCc5cccc(F)c5)c(Cl)c4)c3c2	3.4624	4.1752
390^	CHEMBL513909	521.65	CC[C@H]1N(C2CCCC2)c3nc(Nc4ccc(cc4OC)C(=O)NC5CCN(C)CC5)ncc3N(C)C1=O	-0.0809	1.1034
391	CHEMBL2048912	522.02	CC(C)(N)C(=O)NCCn1ccc2ncnc(Nc3ccc(Oc4cccc5sncc45)c(Cl)c3)c12	4.0000	4.5582
392	CHEMBL1209844	522.54	C[C@@H](Oc1cc(sc1C(=O)N)n2cnc3cc(ccc23)c4ccnc(C)c4)c5ccccc5C(F)(F)F	0.0000	0.4905
393*	CHEMBL1208835	523.53	C[C@@H](Oc1cc(sc1C(=O)N)n2cnc3cc(ccc23)c4ccnc(N)c4)c5ccccc5C(F)(F)F	0.0000	0.4828
394^	CHEMBL1208892	524.52	C[C@@H](Oc1cc(sc1C(=O)N)n2cnc3cc(ccc23)c4ccnc(N)n4)c5ccccc5C(F)(F)F	0.0000	0.4760
395*	CHEMBL1208893	524.52	C[C@@H](Oc1cc(sc1C(=O)N)n2cnc3cc(ccc23)c4cnc(N)nc4)c5ccccc5C(F)(F)F	0.0000	0.4773
396	CHEMBL1964264	525.06	C[C@@H](Oc1cc(sc1C(=O)N)n2cnc3ccc(O[C@H]4CCCN(C)CC4)cc23)c5ccccc5Cl	0.4771	0.7543
397^	CHEMBL1964265	525.06	C[C@@H](Oc1cc(sc1C(=O)N)n2cnc3ccc(O[C@@H]4CCCN(C)CC4)cc23)c5ccccc5Cl	0.6021	0.7543
398*	CHEMBL1794069	525.06	C[C@@H](Oc1cc(sc1C(=O)N)n2cnc3ccc(OCC4CCN(C)CC4)cc23)c5ccccc5Cl	0.7782	0.7545
399^	CHEMBL1808338	525.6	CCc1cccc(CC)c1NC(=O)c2nn(C)c3c2CCc4cnc(Nc5ccc(cc5OC)C(=O)N)nc34	3.4820	3.1176
400	CHEMBL1689168	526.55	CC(Oc1cc(ccc1C(=O)N)n2cnc3cc(OCCCN(C)C)ccc23)c4ccccc4C(F)(F)F	4.1818	3.1646
401*	CHEMBL2392551	527.61	CN1CCC(CC1)NC(=O)c2ccc(Nc3ncc4N(C)C(=O)C(F)(F)CN(C5CCCC5)c4n3)cc2C	2.2788	2.9474
402*	CHEMBL103667	527.66	Cc1ccc(cc1)n2nc(cc2NC(=O)Nc3ccc(OCCN4CCOCC4)c5ccccc35)C(C)(C)C	4.4771	4.4828
403^	CHEMBL1271535	530.55	CN1CCCC1CCNc2ccc(OC(F)(F)F)c(Nc3ncc4CCc5c(nn(C)c5c4n3)C(=O)N)c2	2.1335	1.5154
404*	CHEMBL1964263	530.56	C[C@@H](Oc1cc(sc1C(=O)N)n2cnc3ccc(OC4CCNCC4)cc23)c5ccccc5C(F)(F)F	0.4771	0.4901
405	CHEMBL1614933	530.56	CN1CCC(CC1)Oc2ccc3ncn(c4cc(OCc5ccccc5C(F)(F)F)c(s4)C(=O)N)c3c2	0.9542	0.7530
406	CHEMBL597754	530.64	CNC(=O)c1nn(C)c2c1CCc3cnc(NC4CCN(CC4)C(=O)C5CCN(CC5)S(=O)(=O)C)nc23	4.0000	3.1913
407^	CHEMBL1630107	530.93	ONC(=O)\C=C\c1oc(cc1)c2ccc3ncnc(Nc4ccc(OCc5cccc(F)c5)c(Cl)c4)c3c2	4.0792	4.0647
408	CHEMBL1774337	531.53	CN1CCN(CC1)c2ccc(OC(F)(F)F)c(Nc3ncc4CCc5c(nn(CCN)c5c4n3)C(=O)N)c2	1.5185	1.4553
409*	CHEMBL1933584	531.53	CN1CCN(CC1)c2ccc(OC(F)(F)F)c(Nc3nccc(n3)c4cc5C(=O)NCCc5n4CCO)c2	2.0086	2.2006
410^	CHEMBL1738758	532.52	CN1CCN(CC1)c2ccc(OC(F)(F)F)c(Nc3ncc4CCc5c(nn(CCO)c5c4n3)C(=O)N)c2	0.3010	1.3889
411	CHEMBL1774334	532.52	CN1CCN([C@@H](CO)C1)c2ccc(OC(F)(F)F)c(Nc3ncc4CCc5c(nn(C)c5c4n3)C(=O)N)c2	2.1875	1.4672
412*	CHEMBL1774335	532.52	CN1CCN([C@H](CO)C1)c2ccc(OC(F)(F)F)c(Nc3ncc4CCc5c(nn(C)c5c4n3)C(=O)N)c2	2.3032	1.4672
413^	CHEMBL1774331	532.56	CCN(CC)CCN(C)c1ccc(OC(F)(F)F)c(Nc2ncc3CCc4c(nn(C)c4c3n2)C(=O)N)c1	1.7782	1.5143
414	CHEMBL1208963	534.03	C[C@@H](Oc1cc(sc1C(=O)N)n2cnc3cc(ccc23)c4ccnc(NCCO)c4)c5ccccc5Cl	0.0000	0.7400
415	CHEMBL1271694	534.49	Cn1nc(C(=O)N)c2CCc3cnc(Nc4cc(ccc4OC(F)(F)F)[N+]5([O-])CC[N+](C)([O-])CC5)nc3c12	3.4285	1.4402
416*	CHEMBL1793892	534.51	CN1CCN(CC1)c2ccc(OC(F)(F)F)c(Nc3ncc4CCc5c(nn(CCF)c5c4n3)C(=O)N)c2	0.6021	1.3012
417	CHEMBL2042031	534.57	CC(Oc1cc(ccc1C(=O)N)c2cc(cnc2N)c3ccc(CN(C)C)cc3)c4ccccc4C(F)(F)F	3.1931	3.1587
418^	CHEMBL2042032	534.57	CC(Oc1cc(ccc1C(=O)N)c2cc(cnc2N)c3cccc(CN(C)C)c3)c4ccccc4C(F)(F)F	3.5172	3.1583

419	CHEMBL3218001	534.59	Nc1ccccc1NC(=O)\C=C\c2ccn(c2)S(=O)(=O)c3ccc4ncnc(Nc5cccc(c5)C#C)c4c3	4.4314	4.1865
420	CHEMBL3408947	534.65	COc1ccc2NC(=O)[C@@]3(C[C@H]3c4ccc5c(\C=C\c6ccc(CN7C[C@@H](C)O[C@@H](C)C7)cc6)n[nH]c5c4)c2c1	4.6990	4.6941
421*	CHEMBL2042029	535.56	CC(Oc1cc(ccc1C(=O)N)c2nc(cnc2N)c3ccc(CN(C)C)cc3)c4ccccc4C(F)(F)F	3.5911	3.1405
422*	CHEMBL2042030	535.56	CC(Oc1cc(ccc1C(=O)N)c2nc(cnc2N)c3cccc(CN(C)C)c3)c4ccccc4C(F)(F)F	4.0828	3.1401
423	CHEMBL1808337	536.55	CCc1cccc(CC)c1NC(=O)c2nn(C)c3c2CCc4cnc(Nc5ccccc5OC(F)(F)F)nc34	4.0000	4.3329
424	CHEMBL2046883	537.66	Cc1ccc(NC(=O)Nc2nnc(Sc3ncnc4cc(OCCCN5CCOCC5)ccc34)s2)cc1	4.0000	3.6746
425	CHEMBL1689164	538.56	CC(Oc1cc(ccc1C(=O)N)n2cnc3ccc(OC4CCN(C)CC4)cc23)c5ccccc5C(F)(F)F	2.2788	3.1640
426	CHEMBL1615277	538.56	C[C@@H](Oc1cc(ccc1C(=O)N)n2cnc3cc(OC4CCN(C)CC4)ccc23)c5ccccc5C(F)(F)F	4.7101	3.1666
427*	CHEMBL1689165	538.56	CC(Oc1cc(ccc1C(=O)N)n2cnc3cc(OC4CCN(C)CC4)ccc23)c5ccccc5C(F)(F)F	4.9440	3.1666
428	CHEMBL502835	539.62	COC(=O)c1ccc2\C(=C(\Nc3ccc(cc3)N(C)C(=O)CN4CCN(C)CC4)/c5ccccc5)\C(=O)Nc2c1	4.0000	3.6895
429^	CHEMBL3217993	540.41	Nc1ccccc1NC(=O)c2ccc(COc3ccc4ncnc(Nc5cccc(Br)c5)c4c3)cc2	5.0000	4.2655
430*	CHEMBL2042035	540.6	CC(Oc1cc(ccc1C(=O)N)c2cc(cnc2N)c3ccc(CN(C)C)s3)c4ccccc4C(F)(F)F	2.6990	3.1705
431	CHEMBL2042136	540.6	C[C@@H](Oc1cc(ccc1C(=O)N)c2cc(cnc2N)c3cc(CN(C)C)cs3)c4ccccc4C(F)(F)F	2.9138	3.1705
432*	CHEMBL2042040	540.6	CC(Oc1cc(ccc1C(=O)N)c2cc(cnc2N)c3cc(CN(C)C)cs3)c4ccccc4C(F)(F)F	3.1206	3.1705
433	CHEMBL1630109	540.97	ONC(=O)\C=C\c1cccc(c1)c2ccc3ncnc(Nc4ccc(OCc5cccc(F)c5)c(Cl)c4)c3c2	3.8325	4.0894
434*	CHEMBL1630110	540.97	ONC(=O)\C=C\c1ccc(cc1)c2ccc3ncnc(Nc4ccc(OCc5cccc(F)c5)c(Cl)c4)c3c2	3.9590	4.0934
435^	CHEMBL1964262	541.06	C[C@@H](Oc1cc(sc1C(=O)N)n2cnc3ccc(OC[C@H](O)CN4CCCC4)cc23)c5ccccc5Cl	0.4771	0.7358
436*	CHEMBL1208833	542.96	C[C@@H](Oc1cc(sc1C(=O)N)n2cnc3cc(ccc23)c4ccnc(Cl)c4)c5ccccc5C(F)(F)F	0.6021	0.4924
437	CHEMBL1208834	542.96	C[C@@H](Oc1cc(sc1C(=O)N)n2cnc3ccc(cc23)c4ccnc(Cl)c4)c5ccccc5C(F)(F)F	1.7243	0.4898
438	CHEMBL1933577	543.46	CN1CCN(CC1)c2ccc(OC(F)(F)F)c(Nc3nccc(n3)c4cc(cn4CC(F)(F)F)C(=O)N)c2	1.2553	1.5280
439	CHEMBL2392553	543.61	COc1cc(ccc1Nc2ncc3N(C)C(=O)C(F)(F)CN(C4CCCC4)c3n2)C(=O)NC5CCN(C)CC5	0.6021	0.3744
440*	CHEMBL2392550	543.61	COc1cc(Nc2ncc3N(C)C(=O)C(F)(F)CN(C4CCCC4)c3n2)ccc1C(=O)NC5CCN(C)CC5	1.1761	2.9320
441^	CHEMBL1774336	544.53	CN1CCC(CC1)C(=O)Nc2ccc(OC(F)(F)F)c(Nc3ncc4CCc5c(nn(C)c5c4n3)C(=O)N)c2	2.3502	1.4943
442	CHEMBL1615278	544.59	C[C@@H](Oc1cc(sc1C(=O)N)n2cnc3ccc(OC4CCN(C)CC4)cc23)c5ccccc5C(F)(F)F	0.3010	0.4907
443^	CHEMBL1689158	544.59	CC(Oc1cc(sc1C(=O)N)n2cnc3ccc(OC4CCN(C)CC4)cc23)c5ccccc5C(F)(F)F	0.9542	0.4907
444	CHEMBL1258913	544.67	O=C(N[C@H](CN1CCCC1)c2ccccc2)c3cc4[nH]nc(NC(=O)c5ccc(cc5)N6CCOCC6)c4s3	4.0000	3.6366
445*	CHEMBL1774339	545.56	CN1CCN(CC1)c2ccc(OC(F)(F)F)c(Nc3ncc4CCc5c(nn(CCCN)c5c4n3)C(=O)N)c2	1.7160	1.4828
446*	CHEMBL1793894	546.54	COCCn1nc(C(=O)N)c2CCc3cnc(Nc4cc(ccc4OC(F)(F)F)N5CCN(C)CC5)nc3c12	1.1761	1.4102
447*	CHEMBL1630108	547	ONC(=O)\C=C\c1ccc(s1)c2ccc3ncnc(Nc4ccc(OCc5cccc(F)c5)c(Cl)c4)c3c2	3.4314	4.1177

448^	CHEMBL2392556	547.69	COc1cc(ccc1Nc2ncc3N(C)C(=O)[C@@H](CC=C)CN(C4CCCC4)c3n2)C(=O)NC5CCN(C)CC5	0.0000	1.1616
449	CHEMBL2392557	547.69	COc1cc(ccc1Nc2ncc3N(C)C(=O)[C@H](CC=C)CN(C4CCCC4)c3n2)C(=O)NC5CCN(C)CC5	1.8451	1.1616
450	CHEMBL1614725	547.96	CC(C)(O)CC(=O)NCCn1ccc2ncnc(Nc3ccc(Oc4cccc(c4)C(F)(F)F)c(Cl)c3)c12	4.0000	4.5155
451*	CHEMBL1209017	549.09	C[C@@H](Oc1cc(sc1C(=O)N)n2cnc3cc(ccc23)c4cnn(CCCN(C)C)c4)c5ccccc5Cl	0.3010	0.7515
452*	CHEMBL1793895	550.96	CN1CCN(CC1)c2ccc(OC(F)(F)F)c(Nc3ncc4CCc5c(nn(CCCl)c5c4n3)C(=O)N)c2	0.8451	1.5302
453*	CHEMBL2392558	551.66	COc1cc(ccc1Nc2ncc3N(C)C(=O)[C@](F)(CN(C4CCCC4)c3n2)C=C)C(=O)NC5CCN(C)CC5	0.6990	1.6288
454^	CHEMBL2392543	551.66	COc1cc(ccc1Nc2ncc3N(C)C(=O)[C@@](F)(CN(C4CCCC4)c3n2)C=C)C(=O)NC5CCN(C)CC5	2.2304	1.6288
455	CHEMBL2392544	552.64	COc1cc(ccc1Nc2ncc3N(C)C(=O)[C@](F)(CN(C4CCCC4)c3n2)C=C)C(=O)NN5CCN(C)CC5	0.4771	1.6247
456	CHEMBL2392554	553.67	CC[C@@]1(F)CN(C2CCCC2)c3nc(Nc4ccc(cc4OC)C(=O)NC5CCN(C)CC5)ncc3N(C)C1=O	0.3010	1.7207
457*	CHEMBL2392555	553.67	CC[C@]1(F)CN(C2CCCC2)c3nc(Nc4ccc(cc4OC)C(=O)NC5CCN(C)CC5)ncc3N(C)C1=O	2.0000	1.7207
458	CHEMBL1929238	554.53	CN(C)CCN1CCN(CCC1=O)C(=O)c2cc(sc2NC(=O)Nc3cccc(Cl)c3Cl)C(C)(C)C	4.4771	4.5414
459	CHEMBL1272250	556.58	Cn1nc(C(=O)N)c2CCc3cnc(Nc4cc(ccc4OC(F)(F)F)N5CCC(CC5)N6CCCC6)nc3c12	1.5185	1.5186
460*	CHEMBL1794068	558.62	C[C@@H](Oc1cc(sc1C(=O)N)n2cnc3ccc(OCC4CCN(C)CC4)cc23)c5ccccc5C(F)(F)F	0.8451	0.4914
461*	CHEMBL1208894	559.08	C[C@@H](Oc1cc(sc1C(=O)N)n2cnc3cc(ccc23)c4ccnc(c4)N5CCNCC5)c6ccccc6Cl	0.9031	0.7500
462*	CHEMBL1774333	559.59	CN1CCN(CCCNc2ccc(OC(F)(F)F)c(Nc3ncc4CCc5c(nn(C)c5c4n3)C(=O)N)c2)CC1	2.0253	1.5137
463	CHEMBL1834657	560.48	CCN1CCN(CC1)c2ccc(Nc3cc(ncn3)N(C)C(=O)Nc4c(Cl)c(OC)cc(OC)c4Cl)cc2	4.0000	3.7751
464	CHEMBL1983268	560.64	CN1CCN(CC1)c2ccc(C(=O)Nc3n[nH]c4ccc(Cc5cc(F)cc(F)c5)cc34)c(NC6CCOCC6)c2	4.0000	3.7174
465	CHEMBL1933581	561.56	Cn1cc(cc1c2ccnc(Nc3cc(ccc3OC(F)(F)F)N4CCN(CC4)C(=O)OC(C)(C)C)n2)C(=O)N	4.0000	2.9075
466^	CHEMBL2392545	561.6	COc1cc(C(=O)NC2CCN(C)CC2)c(F)cc1Nc3ncc4N(C)C(=O)C(F)(F)CN(C5CCCC5)c4n3	0.3010	0.3388
467*	CHEMBL2392547	561.6	COc1c(F)c(ccc1Nc2ncc3N(C)C(=O)C(F)(F)CN(C4CCCC4)c3n2)C(=O)NC5CCN(C)CC5	1.4150	0.3388
468*	CHEMBL2392546	561.6	COc1cc(cc(F)c1Nc2ncc3N(C)C(=O)C(F)(F)CN(C4CCCC4)c3n2)C(=O)NC5CCN(C)CC5	1.9085	0.3310
469	CHEMBL2148053	562.47	CC(C)(C(=O)NCCn2ccc3ncnc(Nc4ccc(Oc5cccc(Cl)c5)c(Cl)c4)c23)S(=O)(=O)C	4.0000	4.4105
470	CHEMBL1933583	569.5	CN1CCN(CC1)c2ccc(OC(F)(F)F)c(Nc3nccc(n3)c4ccc5C(=O)NCCc5n4CC(F)(F)F)c2	1.4472	1.7330
471^	CHEMBL220241	570.6	CCn1c(nc2cnc(Oc3cccc(NC(=O)c4ccc(OCCN5CCOCC5)cc4)c3)cc12)c6nonc6N	4.0000	3.6570

472	CHEMBL1774329	572.63	CN(C)CCCN1CCC(CC1)c2ccc(OC(F)(F)F)c(Nc3ncc4CCc5c(nn(C)c5c4n3)C(=O)N)c2	1.6232	1.5205
473	CHEMBL1271482	572.63	CN1C(C)(C)CC(CC1(C)C)Nc2ccc(OC(F)(F)F)c(Nc3ncc4CCc5c(nn(C)c5c4n3)C(=O)N)c2	2.9841	2.3922
474	CHEMBL1684800	572.97	COc1ccc(Cn2ncc(NC(=O)c3cc(NC(=O)Nc4ccc(Cl)c(c4)C(F)(F)F)ccc3C)c2N)cc1	2.2238	4.0150
475^	CHEMBL1208958	573.11	C[C@@H](Oc1cc(sc1C(=O)N)n2cnc3cc(ccc23)c4ccnc(c4)N5CCN(C)CC5)c6ccccc6Cl	0.3010	0.7503
476^	CHEMBL1774340	573.61	CN(C)CCCn1nc(C(=O)N)c2CCc3cnc(Nc4cc(ccc4OC(F)(F)F)N5CCN(C)CC5)nc3c12	2.4456	1.4935
477^	CHEMBL1209015	577.1	C[C@@H](Oc1cc(sc1C(=O)N)n2cnc3cc(ccc23)c4cnn(CCN5CCOCC5)c4)c6ccccc6Cl	0.0000	0.7481
478*	CHEMBL2392549	578.05	COc1cc(C(=O)NC2CCN(C)CC2)c(Cl)cc1Nc3ncc4N(C)C(=O)C(F)(F)CN(C5CCCC5)c4n3	0.0000	0.3773
479	CHEMBL2443026	578.66	CN(C)c1ccc(Oc2cc(O)cc(O)c2c3onc(c3)C(=O)NC4CCN(CC4)C5CCC6(CC5)OCCO6)cc1	1.0000	2.0062
480^	CHEMBL1808339	579.57	CCc1cccc(CC)c1NC(=O)c2nn(C)c3c2CCc4cnc(Nc5ccc(cc5OC(F)(F)F)C(=O)N)nc34	4.0000	2.9203
481*	CHEMBL1630111	580.01	Nc1ccccc1NC(=O)c2oc(cc2)c3ccc4ncnc(Nc5ccc(OCc6cccc(F)c6)c(Cl)c5)c4c3	3.5911	4.1820
482	CHEMBL554	581.06	CS(=O)(=O)CCNCc1oc(cc1)c2ccc3ncnc(Nc4ccc(OCc5cccc(F)c5)c(Cl)c4)c3c2	5.0000	3.8697
483	CHEMBL1208964	587.13	C[C@@H](Oc1cc(sc1C(=O)N)n2cnc3cc(ccc23)c4ccnc(NC5CCN(C)CC5)c4)c6ccccc6Cl	0.0000	0.7499
484*	CHEMBL1208965	587.13	C[C@@H](Oc1cc(sc1C(=O)N)n2cnc3cc(ccc23)c4ccc(NC5CCN(C)CC5)nc4)c6ccccc6Cl	0.3010	0.7495
485^	CHEMBL1090360	587.69	O=C(Cc1ccccc1)Nc2cccc(c2)c3nc4sccn4c3c5ccnc(Nc6cccc(c6)N7CCOCC7)n5	3.0000	3.8000
486*	CHEMBL1208966	588.12	C[C@@H](Oc1cc(sc1C(=O)N)n2cnc3cc(ccc23)c4ccnc(NC5CCN(C)CC5)n4)c6ccccc6Cl	0.0000	0.7431
487^	CHEMBL1269487	588.58	Cn1nc(C(=O)N)c2CCc3cnc(Nc4cc(ccc4OC(F)(F)F)N5CCN(CC5)C(=O)OC(C)(C)C)nc3c12	2.9340	2.3458
488^	CHEMBL1933580	589.61	CN1CCN(CC1)c2ccc(OC(F)(F)F)c(Nc3nccc(n3)c4cc(cn4CCOC5CCCCO5)C(=O)N)c2	2.4346	1.9228
489	CHEMBL1209016	590.14	C[C@@H](Oc1cc(sc1C(=O)N)n2cnc3cc(ccc23)c4cnn(CCN5CCN(C)CC5)c4)c6ccccc6Cl	0.0000	0.7510
490	CHEMBL1208960	593.62	C[C@@H](Oc1cc(sc1C(=O)N)n2cnc3cc(ccc23)c4ccnc(c4)N5CCOCC5)c6ccccc6C(F)(F)F	0.6990	0.4835
491^	CHEMBL1269490	594.59	COc1ccc(Cn2nc(C(=O)N)c3CCc4cnc(Nc5cc(ccc5OC(F)(F)F)N6CCNCC6)nc4c23)cc1	3.4232	1.4714
492*	CHEMBL1208961	594.65	C[C@@H](Oc1cc(sc1C(=O)N)n2cnc3cc(ccc23)c4ccnc(NCCN(C)C)c4)c5ccccc5C(F)(F)F	0.4771	0.4845
493	CHEMBL1630112	596.07	Nc1ccccc1NC(=O)c2ccc(s2)c3ccc4ncnc(Nc5ccc(OCc6cccc(F)c6)c(Cl)c5)c4c3	3.7482	4.3293

494	CHEMBL1208962	596.12	C[C@@H](Oc1cc(sc1C(=O)N)n2cnc3cc(ccc23)c4ccnc(NCCS(=O)(=O)C)c4)c5ccccc5Cl	0.0000	0.7301
495*	CHEMBL1808341	596.72	CCc1cccc(CC)c1NC(=O)c2nn(C)c3c2CCc4cnc(Nc5ccc(cc5OC)C(=O)NCCN(C)C)nc34	3.2180	3.2671
496	CHEMBL1269488	600.6	Cn1nc(C(=O)N)c2CCc3cnc(Nc4cc(ccc4OC(F)(F)F)N5CCN(CC5)C(=O)N6CCNCC6)nc3c12	1.5441	1.4818
497	CHEMBL1933585	601.62	CN1CCN(CC1)c2ccc(OC(F)(F)F)c(Nc3nccc(n3)c4cc5C(=O)N(CCc5n4C)C(=O)OC(C)(C)C)c2	2.9122	2.8006
498	CHEMBL1630113	606.05	Nc1ccccc1NC(=O)\C=C\c2oc(cc2)c3ccc4ncnc(Nc5ccc(OCc6cccc(F)c6)c(Cl)c5)c4c3	3.9294	4.2188
499	CHEMBL1208959	606.66	C[C@@H](Oc1cc(sc1C(=O)N)n2cnc3cc(ccc23)c4ccnc(c4)N5CCN(C)CC5)c6ccccc6C(F)(F)F	0.4771	0.4872
500^	CHEMBL1630114	616.08	Nc1ccccc1NC(=O)\C=C\c2cccc(c2)c3ccc4ncnc(Nc5ccc(OCc6cccc(F)c6)c(Cl)c5)c4c3	3.7404	4.2435
501	CHEMBL1630115	616.08	Nc1ccccc1NC(=O)\C=C\c2ccc(cc2)c3ccc4ncnc(Nc5ccc(OCc6cccc(F)c6)c(Cl)c5)c4c3	3.7924	4.2474
502	CHEMBL1774338	616.63	CN1CCN(CC1)c2ccc(OC(F)(F)F)c(Nc3ncc4CCc5c(nn(CCOC6CCOCC6)c5c4n3)C(=O)N)c2	1.6335	1.3941
503	CHEMBL1630116	618.1	Nc1ccccc1NC(=O)CCc2ccc(cc2)c3ccc4ncnc(Nc5ccc(OCc6cccc(F)c6)c(Cl)c5)c4c3	4.4314	3.7572
504*	CHEMBL1209018	618.19	CCN1CCN(CCCn2cc(cn2)c3ccc4c(c3)ncn4c5cc(O[C@H](C)c6ccccc6Cl)c(s5)C(=O)N)CC1	0.0000	0.7519
505	CHEMBL1233528	618.81	CC[C@H]1N(C(C)C)c2nc(Nc3ccc(cc3OC)C(=O)N[C@@H]4CC[C@H](CC4)N5CCN(CC6CC6)CC5)ncc2N(C)C1=O	-0.0605	1.1124
506	CHEMBL1808340	622.76	CCc1cccc(CC)c1NC(=O)c2nn(C)c3c2CCc4cnc(Nc5ccc(cc5OC)C(=O)NC6CCN(C)CC6)nc34	2.3747	3.2691
507^	CHEMBL1807305	622.76	CCc1cccc(CC)c1NC(=O)c2nn(C)c3c2CCc4cnc(Nc5ccc(cc5OC)C(=O)N6CCCN(C)CC6)nc34	4.0000	3.2697
508*	CHEMBL1807303	636.79	CCc1cccc(CC)c1NC(=O)c2nn(C)c3c2CCc4cnc(Nc5ccc(cc5OC)C(=O)N6CCC(CC6)N(C)C)nc34	4.0000	3.2698
509*	CHEMBL1808342	650.69	CCc1cccc(CC)c1NC(=O)c2nn(C)c3c2CCc4cnc(Nc5ccc(cc5OC(F)(F)F)C(=O)NCCN(C)C)nc34	4.0000	3.0916
510^	CHEMBL1807301	662.82	CCc1cccc(CC)c1NC(=O)c2nn(C)c3c2CCc4cnc(Nc5ccc(cc5OC)C(=O)N6CCC[C@H]6CN7CCCC7)nc34	4.0000	3.2715
511*	CHEMBL1808343	662.82	CCc1cccc(CC)c1NC(=O)c2nn(C)c3c2CCc4cnc(Nc5ccc(cc5OC)C(=O)N6CCC[C@@H]6CN7CCCC7)nc34	4.0000	3.2715
512	CHEMBL2207846	674.64	CC(C)C[C@H](NC(=O)[C@@H]1CCCN1C(=O)C)C(=O)N[C@@H](Cc2cnc[nH]2)C(=O)N[C@@H](CO)C(=O)N[C@@H]([C@@H](C)OP(=O)(O)O)C(=O)N	4.2041	2.9083

513^	CHEMBL2323729	675.63	CC(C)C[C@H](NC(=O)[C@@H]1CCCN1C(=O)C)C(=O)N[C@@H](Cc2cnc[nH]2)C(=O)N[C@@H](CO)C(=O)N[C@@H]([C@@H](C)OP(=O)(O)O)C(=O)O	3.7218	2.6336
514	CHEMBL1807306	676.73	CCc1cccc(CC)c1NC(=O)c2nn(C)c3c2CCc4cnc(Nc5ccc(cc5OC(F)(F)F)C(=O)N6CCCN(C)CC6)nc34	4.0000	3.1706
515^	CHEMBL1236095	676.73	CCc1cccc(CC)c1NC(=O)c2nn(C)c3c2CCc4cnc(Nc5ccc(cc5OC(F)(F)F)C(=O)NC6CCN(C)CC6)nc34	4.0000	3.1188
516*	CHEMBL1807304	690.76	CCc1cccc(CC)c1NC(=O)c2nn(C)c3c2CCc4cnc(Nc5ccc(cc5OC(F)(F)F)C(=O)N6CCC(CC6)N(C)C)nc34	4.0000	3.1738
517	CHEMBL1807300	716.8	CCc1cccc(CC)c1NC(=O)c2nn(C)c3c2CCc4cnc(Nc5ccc(cc5OC(F)(F)F)C(=O)N6CCC[C@@H]6CN7CCCC7)nc34	4.0000	3.1927
518^	CHEMBL1807302	716.8	CCc1cccc(CC)c1NC(=O)c2nn(C)c3c2CCc4cnc(Nc5ccc(cc5OC(F)(F)F)C(=O)N6CCC[C@H]6CN7CCCC7)nc34	4.0000	3.1927
519	CHEMBL1269489	730.78	CN1CCN(CC1)c2ccc(OC(F)(F)F)c(Nc3ncc4CCc5c(nn(c5c4n3)C(c6ccccc6)(c7ccccc7)c8ccccc8)C(=O)N)c2	4.0000	2.2971
520*	CHEMBL2380765	776.75	CC(C)C[C@@H]1NC(=O)[C@@H]2CCCN2C(=O)CSC[C@H](NC(=O)[C@@H](NC(=O)[C@H](CO)NC(=O)[C@H](Cc3cnc[nH]3)NC1=O)[C@@H](C)OP(=O)(O)O)C(=O)O	3.9912	2.7162
521	CHEMBL2207841	839.91	CC(C)C[C@H](NC(=O)[C@@H]1CCCN1C(=O)C)C(=O)N[C@@H](CCC(=O)NCCCCCCc2ccccc2)C(=O)N[C@@H](CO)C(=O)N[C@@H]([C@@H](C)OP(=O)(O)O)C(=O)N	2.1461	2.9001
522*	CHEMBL2207839	839.91	CC(C)C[C@H](NC(=O)[C@@H]1CCCN1C(=O)C)C(=O)N[C@@H](CCCCNC(=O)CCCCCc2ccccc2)C(=O)N[C@@H](CO)C(=O)N[C@@H]([C@@H](C)OP(=O)(O)O)C(=O)N	2.8129	2.9150
523^	CHEMBL2207842	839.91	CC(C)C[C@H](NC(=O)[C@@H]1CCCN1C(=O)C)C(=O)N[C@@H](CC(=O)NCCCCCCCCc2ccccc2)C(=O)N[C@@H](CO)C(=O)N[C@@H]([C@@H](C)OP(=O)(O)O)C(=O)N	3.0414	2.8873
524	CHEMBL2207840	853.94	CC(C)C[C@H](NC(=O)[C@@H]1CCCN1C(=O)C)C(=O)N[C@@H](CCCNC(=O)CCCCCCCc2ccccc2)C(=O)N[C@@H](CO)C(=O)N[C@@H]([C@@H](C)OP(=O)(O)O)C(=O)N	2.2304	2.9118
525*	CHEMBL2207845	862.95	CC(C)C[C@H](NC(=O)[C@@H]1CCCN1C(=O)C)C(=O)N[C@@H](Cc2cncn2CCCCCCCCc3ccccc3)C(=O)N[C@@H](CO)C(=O)N[C@@H]([C@@H](C)OP(=O)(O)O)C(=O)N	0.7482	2.9072
526	CHEMBL2207844	862.95	CC(C)C[C@H](NC(=O)[C@@H]1CCCN1C(=O)C)C(=O)N[C@@H](Cc2cn(CCCCCCCCc3ccccc3)cn2)C(=O)N[C@@H](CO)C(=O)N[C@@H]([C@@H](C)OP(=O)(O)O)C(=O)N	2.5051	2.9058
527^	CHEMBL2380653	878.89	CC(C)C[C@@H]1NC(=O)CN(CCc2c[nH]c3ccccc23)C(=O)CSC[C@@H](NC(=O)[C@@H](NC(=O)[C@H](CO)NC(=O)[C@H](Cc4c[nH]cn4)NC1=O)[C@@H](C)OP(=O)(O)O)C(=O)N	3.9294	2.8421

528	CHEMBL2207843	912.02	CC(C)C[C@H](NC(=O)[C@@H]1CCCN1C(=O)C)C(=O)N[C@@H](Cc2cn(CCCCCCCCc3ccccc3)c4ccccc24)C(=O)N[C@@H](CO)C(=O)N[C@@H]([C@@H](C)OP(=O)(O)O)C(=O)N	3.9685	2.9063
529	CHEMBL505637	942.24	O\N=C(\Cc1cc(Br)c(O)c(c1)c2cc(C\C(=N\O)\C(=O)NCCc3ccc(O)c(Br)c3)cc(Br)c2O)/C(=O)NCCc4ccc(O)c(Br)c4	3.5911	3.5256
530	CHEMBL2380651	949.97	CC(C)C[C@@H]1NC(=O)CN(CCC(=O)NCCc2c[nH]c3ccccc23)C(=O)CSC[C@@H](NC(=O)[C@@H](NC(=O)[C@H](CO)NC(=O)[C@H](Cc4c[nH]cn4)NC1=O)[C@@H](C)OP(=O)(O)O)C(=O)N	3.6767	2.8256

[a] validation set compound [b] test set compound

Table 2S. List of mathematical equations used in the present study.

$$S = \sqrt{\frac{\sum_{i=1}^{N}\left(p_i^{exp} - p_i^{pred}\right)^2}{N - d - 1}}$$

$$h_i = x_i(\mathbf{X}^T\mathbf{X})^{-1}x_i^T$$

$$h^* = 3(d+1)/N_{train}$$

$$k = \frac{\sum_{i=1}^{N_{test}}\left(p_i^{exp} p_i^{pred}\right)^2}{\sum_{i=1}^{N_{test}}\left(p_i^{pred}\right)^2}$$

$$k' = \frac{\sum_{i=1}^{N_{test}}\left(p_i^{exp} p_i^{pred}\right)^2}{\sum_{i=1}^{N_{test}}\left(p_i^{\mathrm{exp}}\right)^2}$$

$$p_0^{exp} = kp^{pred}$$

$$p_0^{pred} = k' p^{exp}$$

$$R_0^2 = 1 - \frac{\sum_{i=1}^{N_{test}}\left(p_i^{exp} - p_{0i}^{exp}\right)^2}{\sum_{i=1}^{N_{test}}\left(p_i^{exp} - p_{av}^{exp}\right)^2}$$

$$R_0'^2 = 1 - \frac{\sum_{i=1}^{N_{test}}\left(p_i^{pred} - p_{0i}^{pred}\right)^2}{\sum_{i=1}^{N_{test}}\left(p_i^{pred} - p_{av}^{pred}\right)^2}$$

$$R_m^2 = R_{test}^2\left(1 - \left|\sqrt{R_{test}^2 - R_0^2}\right|\right)$$

$$VIF = \frac{1}{1 - R_j^2}$$

S: standard deviation; *N*: number of molecules; p_i^{exp} : experimental activity for compound *i*; p_i^{pred} : predicted activity for compound *i*; *d*: number of descriptors; h_i : leverage for compound *i*; x_i: descriptor vector for *i*; $\mathbf{X}$: model matrix for

the training set (train); N_{train} : number of molecules in train; N_{test} =number of molecules in test; h^* : warning leverage; p_{0i}^{exp} and p_{0i}^{pred} : calculated activities for *i* in test set in regressions through the origin of p^{exp} against p^{pred} and p^{pred} against p^{exp}, respectively; R_0^2 and $R_0^{'2}$: squared correlation coefficients for regressions through the origin in test set of p^{exp} against p^{pred} and p^{pred} against p^{exp}, respectively; p_{av}^{pred} : average value for p^{pred} in test set; p_{av}^{exp} : average value for p^{exp} in test set; R_m^2 : modified squared correlation coefficient; R_{test}^2 : squared correlation coefficient between observed and predicted values for the test set; *VIF*: variance inflation factor; R_j^2 is the squared correlation coefficient when the jth variable is regressed against all the other variables in the model.

Table 3S. A brief description for molecular descriptors appearing in this QSAR study.

**descriptor class**	**symbol**	**description**
2D Atom Pairs Fingerprint	*AP*159	presence of C-O at topological distance 3
	*AP*170	presence of N-O at topological distance 3
	*APC*510	count of S-X at topological distance 7
Klekota Roth Fingerprint	*KR*3577	presence of SMARTS substructure Cc1cccc(C)c1NC=O
	*KRC*3897	number of SMARTS substructures CN(C)CCCNC=O
	*KR*4261	presence of SMARTS substructure NC1CCCC1
	*KR*4268	presence of SMARTS substructure Nc1ccccc1O
Pubchem Fingerprint	*PC*494	presence of O=C-C:N [a]
	*PC*534	presence of S-C:C-O
	*PC*686	presence of O=C-C-C-C-O
Substructure Fingerprint	*Sub*99	presence of primary amides
Hydrogen Bond Acceptor Count	*nHBAcc*3	number of H bond acceptors (any O; any N where the formal charge of the N is non-positive (i.e. formal charge <= 0) except a non-aromatic N that is adjacent to an O and aromatic ring, or an aromatic N with a H atom in a ring, or an aromatic N with 3 neighboring atoms in a ring, or a N with total bond order >=4, or a N in an amide bond; any F)
Electrotopological State Atom Type	*maxHCsats*	maximum atom-type H E-State: H bonded to B, Si, P, Ge, As, Se, Sn or Pb
	mindssC	minimum atom-type E-State: =C<
MACCS Fingerprint	*M*66	number of CC(C)(C)A [b]

[a] denotes bond aromaticity [b] A: any valid periodic table element symbol

Table 4S. Squared correlation matrix for Eq. 1 and the variance inflation factor (*VIF*) for each descriptor.

	mindssC	*maxHCsats*	*M66*	*PC494*	*PC534*	*PC686*	*KR3577*	*KR4268*	*VIF*
mindssC	1	0.04	0.03	0.01	0.01	0.00	0.00	0.04	1.018
maxHCsats		1	0.02	0.04	0.06	0.00	0.01	0.05	1.040
M66			1	0.00	0.01	0.01	0.00	0.02	1.012
PC494				1	0.03	0.00	0.07	0.14	1.049
PC534					1	0.01	0.00	0.02	1.024
PC686						1	0.06	0.04	1.010
KR3577							1	0.13	1.046
KR4268								1	1.107

Table 5S. Numerical descriptor values involved in Eq. 1.

ID	*mindssC*	*maxHCsats*	*M66*	*PC494*	*PC534*	*PC686*	*KR3577*	*KR4268*
1	-0.04324074	0.48743056	0	0	0	0	0	0
2	-0.68435185	0.00000000	0	0	0	1	0	0
3	-0.48654809	0.00000000	0	0	0	0	0	0
4	0.00000000	0.00000000	0	0	0	1	0	0
5	0.01185752	0.55977584	0	0	0	0	0	0
6	-0.79375000	0.00000000	0	0	0	1	0	0
7	-0.04557587	0.57615276	0	0	0	0	0	0
8	-0.09326389	0.00000000	0	0	0	0	0	0
9	-0.11153439	0.00000000	0	0	0	0	0	0
10	-0.13939531	0.00000000	0	0	0	0	0	0
11	1.00310185	0.60027778	0	0	0	0	0	0
12	-0.02823507	0.00000000	0	0	0	0	0	0
13	-0.06823507	0.00000000	0	0	0	0	0	0
14	-0.08864324	0.00000000	0	0	0	0	0	0
15	1.01060185	0.60027778	0	0	0	0	0	0
16	1.00717593	0.60027778	0	0	0	0	0	0
17	-0.19151408	0.00000000	0	0	0	0	0	0
18	0.96474206	0.61315760	0	0	0	0	0	0
19	-0.39699074	0.00000000	0	0	0	0	0	0
20	-0.05695318	0.00000000	0	0	0	0	0	0
21	-0.05934477	0.00000000	0	0	0	0	0	0
22	-0.07496977	0.00000000	0	0	0	0	0	0
23	-0.84145571	0.00000000	0	1	0	0	0	0
24	0.00000000	0.00000000	0	0	0	0	0	0
25	-0.35671937	0.00000000	0	0	1	0	0	0
26	1.03677469	0.59959191	0	0	0	0	0	0
27	1.02535151	0.59977387	0	0	0	0	0	0
28	-0.54949074	0.00000000	0	0	0	1	0	0
29	-0.22193736	0.00000000	0	0	0	0	0	0
30	-0.22193736	0.00000000	0	0	0	0	0	0
31	-0.50149896	0.00000000	0	0	0	0	0	0

32	0.01990741	0.56704436	0	0	0	0	0	0
33	-0.07263039	0.00000000	0	0	0	0	0	0
34	-0.09303855	0.00000000	0	0	0	0	0	0
35	-0.93149749	0.69421485	0	0	0	0	0	0
36	0.98699172	0.61277179	0	0	0	0	0	0
37	0.99841490	0.61265369	0	0	0	0	0	0
38	-0.94149749	0.69421485	0	0	0	0	0	0
39	-0.10374008	0.00000000	0	0	0	0	0	0
40	-0.61013104	0.00000000	0	0	0	0	0	0
41	-0.63053920	0.00000000	0	0	0	0	0	0
42	-0.62575604	0.00000000	0	0	0	0	0	0
43	-0.03709511	0.00000000	0	0	0	0	0	0
44	-0.03709511	0.00000000	0	0	0	0	0	0
45	0.00000000	0.00000000	0	0	0	0	0	0
46	0.00000000	0.00000000	0	0	0	0	0	0
47	0.00000000	0.00000000	0	0	0	0	0	0
48	-0.94088057	0.00000000	0	0	0	1	0	0
49	-0.88767149	0.00000000	0	0	0	0	0	0
50	-0.05225134	0.00000000	0	0	0	0	0	0
51	-0.83285346	0.68161033	0	0	0	0	0	0
52	-0.93012008	0.69421485	0	0	0	0	0	0
53	-0.84111792	0.68161033	0	0	0	0	0	0
54	1.16410777	0.58263590	0	0	0	0	0	0
55	-0.52533320	0.63512706	0	1	0	0	0	0
56	-1.04511217	0.00000000	0	1	0	0	0	0
57	-0.61764214	0.55247629	0	0	0	0	0	0
58	0.00000000	0.00000000	0	0	0	0	0	0
59	0.00000000	0.66577389	0	1	0	0	0	0
60	0.00000000	0.00000000	0	0	0	0	0	0
61	-0.05181133	0.00000000	0	0	0	0	0	0
62	-0.83169605	0.68161033	0	0	0	0	0	0
63	-0.78157478	0.67303607	0	0	0	0	0	0
64	0.00000000	0.00000000	0	0	0	0	0	0
65	0.00000000	0.00000000	0	0	0	0	0	0
66	-0.93656713	0.70810374	0	0	0	0	0	0
67	-0.15770125	0.00000000	0	0	0	0	0	0
68	-0.15770125	0.00000000	0	0	0	0	0	0
69	-0.09146944	0.00000000	0	0	0	0	0	1
70	-0.08473067	0.00000000	0	0	0	0	0	0
71	-0.18483472	0.00000000	0	0	0	0	0	0
72	-0.07958911	0.00000000	0	0	0	0	0	0
73	-0.84558494	0.70938811	0	0	0	0	0	0
74	-0.11250845	0.00000000	0	0	0	0	0	0
75	-0.10736689	0.00000000	0	0	0	0	0	0
76	-0.11958911	0.00000000	0	0	0	0	0	0
77	-0.12473067	0.00000000	0	0	0	0	0	0
78	-0.50741653	0.63512706	0	1	0	0	0	0
79	-0.53262759	0.52579398	0	0	0	0	0	0
80	-1.00741653	0.65512706	0	1	0	0	0	0
81	-0.40078572	0.00000000	0	0	0	0	0	0
82	0.00000000	0.65705282	0	1	0	0	0	0

83	-0.43420512	0.00000000	0	0	0	0	0	0
84	-0.44983012	0.00000000	0	0	0	0	0	0
85	-0.45461328	0.00000000	0	0	0	0	0	0
86	-0.62710397	0.00000000	0	0	0	0	0	0
87	-0.43663465	0.72606392	0	0	0	1	0	0
88	-0.03908429	0.00000000	0	0	0	0	0	0
89	0.05946145	0.00000000	0	0	0	0	0	0
90	-0.07908429	0.00000000	0	0	0	0	0	0
91	-0.06686207	0.00000000	0	0	0	0	0	0
92	0.00000000	0.65741221	0	0	0	0	0	0
93	0.00000000	0.66577389	0	1	0	0	0	0
94	0.00000000	0.66577389	0	1	0	0	0	0
95	0.00000000	0.66133171	0	1	0	0	0	0
96	-0.78058858	0.67303607	0	0	0	0	0	0
97	0.00000000	0.00000000	0	0	0	0	0	0
98	-0.83714066	0.69549922	0	0	0	0	0	0
99	-0.14238856	0.00000000	0	0	0	0	0	0
100	-0.07880721	0.00000000	0	0	0	0	0	0
101	-0.06420038	0.00000000	0	0	0	0	0	0
102	-0.70700927	0.62620995	0	0	0	0	0	0
103	-0.09197816	0.00000000	0	0	0	0	0	0
104	-0.10420038	0.00000000	0	0	0	0	0	0
105	0.00000000	0.00000000	0	0	0	0	0	0
106	-0.50683802	0.63512706	0	1	0	0	0	0
107	-0.16945357	0.63512706	0	1	0	0	0	0
108	-0.52336694	0.64012706	0	1	0	0	0	0
109	0.00000000	0.65835848	0	1	0	0	0	0
110	0.00000000	0.64418651	0	1	0	0	0	0
111	0.00000000	0.00000000	0	0	0	0	0	0
112	-0.04492623	0.00000000	0	0	0	0	0	0
113	-0.04726743	0.00000000	0	0	0	0	0	0
114	0.00000000	0.00000000	0	0	0	0	0	0
115	-0.55642479	0.65012706	0	1	0	0	0	0
116	-0.43605614	0.72606392	0	0	0	1	0	0
117	-0.96401197	0.00000000	0	1	0	0	0	0
118	1.18635743	0.58225009	0	0	0	0	0	0
119	-0.51831635	0.73192330	0	0	0	1	0	0
120	-0.09865764	0.68242284	0	0	0	0	0	0
121	0.00000000	0.66577389	0	1	0	0	0	0
122	0.00000000	0.59414568	0	0	0	0	0	0
123	-0.05652618	0.69610639	1	1	0	0	0	0
124	-0.06294248	0.59625504	0	0	0	0	0	0
125	-0.11492770	0.00000000	0	0	0	0	0	0
126	0.00000000	0.00000000	0	0	0	0	0	0
127	-0.09072026	0.59921362	0	0	0	0	0	0
128	-0.10294248	0.59972726	0	0	0	0	0	0
129	-0.12398442	0.00000000	0	0	0	0	0	0
130	-0.12912599	0.00000000	0	0	0	0	0	0
131	-0.09667972	0.00000000	0	0	0	0	0	0
132	-0.49926838	0.63814152	0	1	0	0	0	0
133	-0.51328506	0.64012706	0	1	0	0	0	0

134	-0.38473134	0.68153342	0	1	0	0	0	0
135	0.00000000	0.66552697	0	1	0	0	0	0
136	-0.52154953	0.64512706	0	1	0	0	0	1
137	-0.51936558	0.64339153	0	1	0	0	0	0
138	-0.51765052	0.64207151	0	1	0	0	0	0
139	0.00000000	0.00000000	0	0	0	0	0	0
140	-0.45117804	0.00000000	0	0	0	0	0	0
141	-0.07360298	0.00000000	0	0	0	0	0	0
142	-0.10138076	0.00000000	0	0	0	0	0	0
143	-0.11360298	0.00000000	0	0	0	0	0	0
144	-0.45952478	0.73050837	0	0	0	1	0	0
145	-0.45211908	0.72997017	0	0	0	1	0	0
146	-0.08347960	0.00000000	0	0	0	0	0	0
147	0.00000000	0.00000000	0	0	0	1	0	0
148	-0.43064852	0.72596747	0	0	0	1	0	0
149	0.00000000	0.68403835	0	1	0	0	0	0
150	-0.07454616	0.63512706	0	1	0	0	0	0
151	-0.03499967	0.57387633	1	0	0	0	0	0
152	-0.10859570	0.00000000	0	0	0	0	0	0
153	-0.53819228	0.64587374	0	1	0	0	0	0
154	-0.53401328	0.64425963	0	1	0	0	0	0
155	-0.54343505	0.64795764	0	1	0	0	0	0
156	-0.50595800	0.63512706	0	1	0	0	0	0
157	0.00000000	0.65716530	0	0	0	0	0	0
158	-0.01555143	0.78360189	1	1	0	0	0	0
159	-0.55732394	0.65208987	0	1	0	0	0	0
160	0.00000000	0.58050822	0	0	0	0	0	0
161	-0.07186008	0.00000000	0	0	0	0	0	0
162	-0.07777723	0.00000000	0	0	0	0	0	0
163	-0.07880452	0.00000000	0	0	0	0	0	0
164	-0.07696212	0.00000000	0	0	0	0	0	0
165	-0.58091209	0.66078822	0	1	0	0	0	0
166	-0.56149587	0.65339219	0	1	0	0	0	0
167	-0.57009289	0.65662041	0	1	0	0	0	0
168	-0.68773931	0.62793717	0	0	0	0	0	0
169	0.00000000	0.00000000	0	0	0	0	0	0
170	-0.49798050	0.63364558	0	1	0	0	0	0
171	0.00000000	0.00000000	0	0	0	0	0	0
172	-0.55625360	0.73666757	0	0	0	1	0	0
173	-0.63977374	0.73972605	0	0	0	1	0	0
174	-0.59037087	0.73805177	0	0	0	1	0	0
175	-0.51394841	0.63124665	0	1	0	0	0	0
176	-0.50492760	0.55125000	1	0	0	1	0	0
177	-0.79695389	0.00000000	0	0	0	1	0	0
178	0.00000000	0.68403835	0	1	0	0	0	0
179	-0.67953330	0.51629411	1	0	0	0	0	0
180	-0.10733780	0.60069948	0	0	0	0	0	0
181	0.07653280	0.76991873	0	0	0	0	0	0
182	-0.30753312	0.00000000	0	0	0	0	0	0
183	-0.54450082	0.65087374	0	1	0	0	0	0
184	-0.14107504	0.00000000	0	0	0	0	0	0

185	-0.55582410	0.65622211	0	1	0	0	0	0
186	0.00000000	0.63545738	0	0	0	0	0	0
187	-0.07288804	0.00000000	0	0	0	0	0	0
188	0.11979714	0.65664361	1	0	0	0	0	0
189	-0.33237397	0.00000000	0	0	0	0	0	0
190	-0.18399915	0.00000000	0	0	0	0	0	0
191	-0.08076646	0.00000000	0	0	0	0	0	0
192	-0.04531699	0.00000000	0	0	0	0	0	0
193	-0.04211759	0.00000000	0	0	0	0	0	0
194	-0.53141549	0.67312629	0	1	0	0	0	0
195	-0.11799830	0.00000000	0	0	0	0	0	0
196	0.08299602	0.76607185	0	0	0	0	0	0
197	1.53925540	0.58125000	1	1	0	1	0	0
198	0.00000000	0.83509181	0	0	0	0	0	0
199	0.00000000	0.60779768	0	0	0	0	0	0
200	-0.36941488	0.64134840	0	0	1	0	0	0
201	-0.01605257	0.00000000	0	0	0	0	0	0
202	-0.86359756	1.13814152	0	1	0	0	0	0
203	-0.61419423	0.66637706	0	1	0	0	0	0
204	-0.63258411	0.67231715	0	1	0	0	0	0
205	-0.59957501	0.66175428	0	1	0	0	0	0
206	-0.75937062	0.65666485	0	0	0	0	0	0
207	-0.46208372	0.63814152	0	1	0	0	0	0
208	-0.06121283	0.63512706	0	1	0	0	0	0
209	0.17367068	0.67464515	0	0	0	0	0	0
210	-0.06661699	0.46892982	0	0	0	0	0	0
211	0.00000000	0.77912501	0	0	0	0	0	0
212	-0.25617052	0.66245049	0	1	0	0	0	0
213	0.00000000	0.60480035	0	0	0	0	0	0
214	-0.12217255	0.00000000	0	0	0	0	0	0
215	-0.12135743	0.00000000	0	0	0	0	0	0
216	-0.00955365	0.60901595	1	0	0	0	0	0
217	0.00719650	0.00000000	0	0	0	0	0	0
218	-0.81030601	0.63759968	0	0	0	0	0	0
219	-0.51435828	0.64671081	0	1	0	0	0	0
220	-0.21000912	0.66317451	0	1	0	0	0	0
221	0.48049449	0.48845947	1	0	0	0	0	0
222	0.00000000	0.76723773	0	0	0	0	0	0
223	-0.28937294	0.59688281	0	0	0	0	0	0
224	0.00000000	0.51263251	1	0	0	0	0	0
225	0.49851108	0.47641298	1	0	0	0	0	0
226	-0.05471246	0.00000000	0	0	0	0	0	0
227	-0.58326517	0.65621675	0	1	0	0	0	0
228	0.00000000	0.50362422	1	0	0	0	0	0
229	0.12579206	0.76991873	0	0	0	0	0	0
230	-0.47401144	0.66939152	0	1	0	0	0	0
231	-0.62876723	0.67637706	0	1	0	0	0	1
232	0.07023651	0.80991873	0	0	0	0	0	0
233	0.07023651	0.80991873	0	0	0	0	0	0
234	0.11197412	0.60901595	1	0	0	0	0	0
235	-0.94613856	0.00000000	0	0	0	0	0	0

236	0.00197750	0.87509963	0	0	0	0	0	0
237	0.00000000	0.60480035	0	0	0	0	0	0
238	0.00000000	0.67144413	0	1	0	0	0	0
239	0.03044635	0.82033540	0	0	0	0	0	0
240	-0.51153340	0.64489949	0	1	0	0	0	0
241	0.03037823	0.85589095	0	0	0	0	0	0
242	-0.16588592	0.65671444	0	1	0	0	0	0
243	0.48312013	0.48340565	1	0	0	0	0	0
244	-0.51979786	0.64989949	0	1	0	0	0	0
245	0.07669972	0.80607185	0	0	0	0	0	0
246	-0.52806233	0.65489949	0	1	0	0	0	1
247	-0.02517733	0.89589095	0	0	0	0	0	0
248	1.38886710	0.50921064	1	0	0	0	0	0
249	-0.26775091	0.72034490	0	0	0	0	0	0
250	-0.45260026	0.58687500	1	0	0	0	0	0
251	0.38480737	0.52630960	1	0	0	0	0	0
252	-0.00676967	0.88208115	0	0	0	0	0	0
253	-0.76460210	0.65666485	0	0	0	0	0	0
254	0.00000000	0.53140200	1	0	0	0	0	0
255	-0.47859939	0.69369708	0	1	0	0	0	0
256	-0.05186847	0.90061318	0	0	0	0	0	0
257	-0.03964625	0.88936318	0	0	0	0	0	0
258	0.00000000	0.00000000	0	0	0	0	0	1
259	-0.64221611	0.68050930	0	1	0	0	0	1
260	-0.54421417	0.00000000	0	0	0	0	0	0
261	-0.07302754	0.00000000	0	0	0	0	0	0
262	-0.29287939	0.67761812	0	1	0	0	0	0
263	0.00906367	0.87509963	0	0	0	0	0	0
264	0.03507597	0.82033540	0	0	0	0	0	0
265	-0.54994785	0.65773007	0	1	0	0	0	0
266	-0.14081364	0.65227227	0	1	0	0	0	0
267	0.09484031	0.79384963	0	0	0	0	0	0
268	0.00000000	0.63524034	0	0	0	0	0	0
269	-0.37638989	0.79177237	0	0	1	0	0	0
270	-0.04503972	0.00000000	0	0	0	0	0	0
271	-0.58138591	0.96125461	1	0	0	1	0	0
272	-0.50449330	0.64341801	0	1	0	0	0	0
273	-0.08878108	0.53016002	0	0	0	0	0	0
274	-0.27673156	0.94330370	0	0	0	0	0	0
275	-0.58870406	0.96331558	0	0	1	0	0	0
276	-0.51896826	0.63847575	0	1	0	0	0	0
277	0.00000000	0.54983876	1	0	0	0	0	1
278	-0.04478230	0.90061318	0	0	0	0	0	0
279	-0.03256008	0.88936318	0	0	0	0	0	0
280	-0.03501662	0.89498818	0	0	0	0	0	0
281	0.12373172	0.65572241	1	0	0	0	0	0
282	0.00000000	0.60292515	0	0	0	0	0	0
283	-0.04008726	0.00000000	0	0	0	0	0	0
284	0.00000000	0.59861074	0	0	0	0	0	0
285	-0.63388535	0.57852746	0	1	0	0	0	0
286	-0.26329654	0.72370638	0	0	0	0	0	0

287	-0.16555706	0.86366873	0	0	0	0	0	0
288	-0.39077022	0.00000000	0	0	1	0	0	0
289	-0.24215146	0.49472010	0	0	0	1	0	0
290	-0.09693791	0.89691987	0	0	0	0	0	0
291	-0.31189209	0.95217944	0	0	0	0	0	0
292	-0.52022002	0.65181032	0	1	0	0	0	1
293	-0.52048042	0.65082151	0	1	0	0	0	1
294	-0.66576508	0.00000000	0	0	0	0	0	0
295	-0.08093846	0.90132498	0	0	0	0	0	0
296	-0.11742286	0.00000000	0	0	0	0	0	0
297	0.00000000	0.53550303	0	0	0	0	0	0
298	-1.04129894	0.00000000	0	0	0	0	0	1
299	-0.33373723	0.62117797	0	1	0	1	0	0
300	-0.52728758	0.43653696	0	0	0	0	0	0
301	-0.17352764	0.66317451	0	1	0	0	1	0
302	-0.06449836	0.89213670	0	0	0	0	0	0
303	-0.39290882	0.63228908	0	1	0	1	0	0
304	-0.37478408	0.77090948	0	0	0	0	0	0
305	-0.16092743	0.86366873	0	0	0	0	0	0
306	-0.39336281	0.80003683	0	0	1	0	0	0
307	-0.39336281	0.80003683	0	0	1	0	0	0
308	-0.63686317	0.00000000	0	0	0	0	0	0
309	-0.71665953	0.00000000	0	0	0	0	0	0
310	-0.54210554	0.65464089	0	1	0	0	0	0
311	-0.54236594	0.65365209	0	1	0	0	0	0
312	-0.54210554	0.65464089	0	1	0	0	0	0
313	-0.58062638	0.61617120	0	0	0	0	0	1
314	-0.60946280	0.97331558	0	0	1	0	0	0
315	-0.32809579	0.95217944	1	0	0	0	0	0
316	-0.07705472	0.54744941	0	0	0	0	0	0
317	-0.37755221	0.64970522	0	0	1	0	0	0
318	-0.68109142	0.00000000	0	1	0	1	0	0
319	-0.33469365	0.62117797	0	1	0	1	0	0
320	0.00356723	0.93010240	0	0	0	0	0	0
321	-0.83265885	0.00000000	0	0	0	0	0	0
322	-0.23331688	0.00000000	0	0	0	0	0	0
323	0.00000000	0.64217263	0	0	0	0	0	0
324	-0.70376612	0.00000000	0	0	0	0	0	0
325	-0.36954473	0.79409582	0	0	1	0	0	0
326	-0.37185838	0.79631469	0	0	1	0	0	0
327	-0.57970624	0.61617120	0	0	0	0	0	1
328	-0.38014480	0.79586660	0	0	1	0	0	0
329	-1.00886073	0.00000000	0	0	0	0	0	0
330	-0.62384314	0.00000000	0	0	1	0	0	0
331	-0.14081723	0.49355074	0	0	0	0	0	0
332	-0.37057202	0.79470716	0	0	1	0	0	0
333	-0.37317839	0.79708516	0	0	1	0	0	0
334	-0.65251858	0.00000000	0	0	0	0	0	0
335	-0.01989130	0.51741141	0	0	0	0	0	0
336	-0.18766064	0.67317451	0	1	0	0	1	1
337	-0.46860859	0.00000000	0	0	0	0	0	0

338	-0.22897435	0.00000000	0	0	0	0	0	0
339	-0.42321128	0.80279157	0	0	1	0	0	0
340	-0.62835786	0.68306032	0	1	0	0	0	1
341	-0.64535439	0.68306032	0	1	0	0	0	1
342	-0.63108934	0.68306032	0	1	0	0	0	1
343	-0.63174694	0.68908786	0	1	0	0	0	1
344	-0.21869347	0.00000000	0	0	0	0	0	0
345	-0.75166135	0.91972931	0	0	0	0	0	0
346	-0.75166135	0.91972931	0	0	0	0	0	0
347	-0.75166135	0.91972931	0	0	0	0	0	0
348	-0.12145003	0.55021949	0	0	0	0	0	0
349	-0.88287788	0.67488844	0	0	0	0	0	0
350	-0.47226051	0.49798792	1	0	0	0	0	0
351	-0.59886033	0.97354861	0	0	1	0	0	0
352	-0.60514477	0.97625037	0	0	1	0	0	0
353	-0.37422882	0.79432339	0	0	1	0	0	0
354	-0.62808149	0.68273031	0	1	0	0	0	1
355	-0.51169698	0.65741506	0	0	0	0	0	1
356	-0.13873402	0.62255184	0	0	0	0	0	1
357	-1.02501709	1.18658540	0	0	0	0	0	0
358	-0.62743772	0.68306032	0	1	0	0	0	1
359	-0.72626096	0.00000000	0	0	0	0	0	0
360	-0.64351198	0.74163033	0	0	0	0	0	1
361	-0.59670049	0.97308801	0	0	1	0	0	0
362	-0.59798685	0.97489932	0	0	1	0	0	0
363	-0.09667263	0.66269831	0	0	0	1	0	1
364	-0.19514177	0.65932843	0	0	0	0	0	0
365	-0.60261765	0.97563903	0	0	1	0	0	0
366	-0.60364493	0.97604659	0	0	1	0	0	0
367	-0.60493129	0.97785790	0	0	1	0	0	0
368	-0.60625131	0.97837154	0	0	1	0	0	0
369	-0.60496495	0.97656023	0	0	1	0	0	0
370	0.14196326	0.74865673	0	0	0	0	0	0
371	-0.40458297	0.57832751	1	1	0	0	0	0
372	-0.36832665	0.79432339	0	0	1	0	0	0
373	-0.60364493	0.97625037	0	0	1	0	0	0
374	-0.05856340	0.72295639	0	1	0	0	0	0
375	-0.79370513	0.72776121	0	0	0	0	0	0
376	-0.58355577	0.96904758	0	0	1	0	0	0
377	-1.25678832	0.97824551	0	0	0	0	0	0
378	-0.66447476	0.70000476	0	1	0	0	0	1
379	-0.63220893	0.00000000	0	0	0	0	0	0
380	0.00000000	0.00000000	0	0	0	0	0	0
381	-1.08121726	0.00000000	0	0	0	0	0	0
382	-0.65897053	0.75912524	0	1	0	0	0	1
383	-0.61669698	0.68607477	0	1	0	0	0	1
384	-0.62663800	0.68306032	0	1	0	0	0	1
385	-0.62669698	0.68306032	0	1	0	0	0	1
386	-0.62910038	0.68273152	0	1	0	0	0	1
387	-0.64604883	0.68734036	0	1	0	0	0	1
388	-0.01901949	0.00000000	0	0	0	0	0	0

389	-0.40938853	0.00000000	0	0	0	0	0	0
390	-0.09726761	0.77704952	0	0	0	1	0	1
391	-0.18779843	0.70150945	1	0	0	0	0	0
392	-0.60227522	0.97563903	0	0	1	0	0	0
393	-0.61410954	0.97819005	0	0	1	0	0	0
394	-0.62237400	0.98166227	0	0	1	0	0	0
395	-0.62052175	0.98122739	0	0	1	0	0	0
396	-0.36734046	0.79432339	0	0	1	0	0	0
397	-0.36734046	0.79432339	0	0	1	0	0	0
398	-0.36753003	0.79399459	0	0	1	0	0	0
399	-0.54392653	0.68526565	0	1	0	1	1	1
400	-0.75518238	0.99667263	0	0	0	0	0	0
401	-1.25620980	0.97824551	0	0	0	0	0	0
402	-0.33809286	0.70801137	1	0	0	0	0	0
403	-0.62913247	0.68273152	0	1	0	0	0	1
404	-0.60265747	0.97586660	0	0	1	0	0	0
405	-0.59880697	0.66970522	0	0	1	0	0	0
406	-0.19728103	0.63963427	0	1	0	0	0	0
407	-0.64715092	0.00000000	0	0	0	0	0	0
408	-0.65042714	0.74163033	0	1	0	0	0	1
409	-0.20253976	0.74163033	0	0	0	0	0	1
410	-0.69124346	0.79718588	0	1	0	0	0	1
411	-0.64486045	0.73075453	0	1	0	0	0	1
412	-0.64486045	0.73075453	0	1	0	0	0	1
413	-0.63094928	0.68306032	0	1	0	0	0	1
414	-0.38749689	0.80010705	0	0	1	0	0	0
415	-0.67413479	0.74637953	0	1	0	0	0	1
416	-0.73205979	0.87776828	0	1	0	0	0	1
417	-0.77266843	0.99406712	0	0	0	0	0	0
418	-0.77276732	0.99447468	0	0	0	0	0	0
419	-0.38527065	0.00000000	0	0	0	0	0	0
420	0.07792902	0.68696039	1	0	0	0	0	0
421	-0.79307660	1.00427120	0	0	0	0	0	0
422	-0.79317548	1.00467876	0	0	0	0	0	0
423	-0.29487834	0.70442451	0	1	0	0	1	1
424	-0.26660282	0.66488717	0	0	0	0	0	0
425	-0.75263074	0.99873697	0	0	0	0	0	0
426	-0.75088636	0.99667263	0	0	0	0	0	0
427	-0.75088636	0.99667263	0	0	0	0	0	0
428	-0.46948300	0.53683350	0	0	0	0	0	0
429	-0.21529434	0.00000000	0	0	0	0	0	0
430	-0.75693398	0.98875708	0	0	0	0	0	0
431	-0.75693398	0.98875708	0	0	0	0	0	0
432	-0.75693398	0.98875708	0	0	0	0	0	0
433	-0.59395364	0.00000000	0	0	0	0	0	0
434	-0.58553369	0.00000000	0	0	0	0	0	0
435	-0.39636048	0.80024055	0	0	1	0	0	0
436	-0.59840350	0.97551305	0	0	1	0	0	0
437	-0.59994739	0.97771180	0	0	1	0	0	0
438	-0.93588727	1.13051922	0	0	0	0	0	1
439	-1.27376124	0.99059119	0	0	0	1	0	1

440	-1.27092131	0.98824551	0	0	0	0	0	0
441	-0.65670829	0.69242537	0	1	0	0	0	1
442	-0.60139957	0.97586660	0	0	1	0	0	0
443	-0.60139957	0.97586660	0	0	1	0	0	0
444	-0.19695943	0.74737062	0	0	0	0	0	0
445	-0.63586506	0.71732477	0	1	0	0	0	1
446	-0.64543139	0.79718588	0	1	0	0	0	1
447	-0.53307684	0.00000000	0	0	0	0	0	0
448	-0.10215564	0.70610109	0	0	0	1	0	1
449	-0.10215564	0.70610109	0	0	0	1	0	1
450	-0.23945910	0.72347876	1	0	0	0	0	0
451	-0.36963125	0.79643726	0	0	1	0	0	0
452	-0.59625732	0.68333129	0	1	0	0	0	1
453	-0.69042791	0.86732730	1	0	0	1	0	1
454	-0.69042791	0.86732730	1	0	0	1	0	1
455	-0.69382927	0.87028588	1	0	0	1	0	1
456	-0.57237236	0.82392452	1	0	0	1	0	1
457	-0.57237236	0.82392452	1	0	0	1	0	1
458	-0.26957387	0.67662777	1	0	0	0	0	0
459	-0.62229255	0.68273152	0	1	0	0	0	1
460	-0.60060294	0.97553780	0	0	1	0	0	0
461	-0.36927535	0.79837694	0	0	1	0	0	0
462	-0.63009874	0.68427473	0	1	0	0	0	1
463	-0.29146986	0.53344893	0	0	0	0	0	0
464	-0.26806341	0.61397405	0	0	0	0	0	0
465	-0.60468638	0.72318055	1	0	0	0	0	1
466	-1.32276951	1.00559119	0	0	0	1	0	1
467	-1.32276951	1.00559119	0	0	0	1	0	1
468	-1.33297935	1.00910971	0	0	0	1	0	1
469	-0.46484161	0.72367696	1	0	0	0	0	0
470	-0.49491504	1.13051922	0	0	0	0	0	1
471	-0.24300256	0.69846495	0	0	0	0	0	0
472	-0.62235389	0.68047129	0	1	0	0	0	1
473	-0.63208649	0.68273152	1	1	0	0	0	1
474	-0.75401546	0.00000000	0	0	0	0	0	0
475	-0.36847563	0.79837694	0	0	1	0	0	0
476	-0.61284552	0.71732477	0	1	0	0	0	1
477	-0.37144753	0.79943036	0	0	1	0	0	0
478	-1.26777512	0.99034428	0	0	0	1	0	1
479	-0.33462977	0.64586932	0	1	0	1	0	0
480	-0.91084829	0.71651565	0	1	0	1	1	1
481	-0.39487207	0.00000000	0	0	0	0	0	0
482	0.00000000	0.58149228	0	0	0	0	0	0
483	-0.36985885	0.79819005	0	0	1	0	0	0
484	-0.37086653	0.79811064	0	0	1	0	0	0
485	-0.05610583	0.63255514	0	0	0	0	0	0
486	-0.37812331	0.80166227	0	0	1	0	0	0
487	-0.65241787	0.72613913	1	1	0	0	0	1
488	-0.60314863	0.84897475	0	0	0	0	0	1
489	-0.36774126	0.79804532	0	0	1	0	0	0
490	-0.60625452	0.98165025	0	0	1	0	0	0

491	-0.65346812	0.72110776	0	1	0	0	0	1
492	-0.60725161	0.97992015	0	0	1	0	0	0
493	-0.07799964	0.00000000	0	0	0	0	0	0
494	-0.40338759	0.80308133	0	0	1	0	0	0
495	-0.21862764	0.68721878	0	1	0	1	1	1
496	-0.64587465	0.71308092	0	1	0	0	0	1
497	-0.71040799	0.79095462	1	0	0	0	0	1
498	-0.31576203	0.00000000	0	0	0	0	0	0
499	-0.60154855	0.97992015	0	0	1	0	0	0
500	-0.26256475	0.00000000	0	0	0	0	0	0
501	-0.25414480	0.00000000	0	0	0	0	0	0
502	-0.64271768	0.81759405	0	1	0	0	0	1
503	-0.07358924	0.67330214	0	0	0	0	0	0
504	-0.36640478	0.79768726	0	0	1	0	0	0
505	-0.07601108	0.77807650	0	0	0	1	0	1
506	-0.21482361	0.68699575	0	1	0	1	1	1
507	-0.21307933	0.68721878	0	1	0	1	1	1
508	-0.21319595	0.68699575	0	1	0	1	1	1
509	-0.53876496	0.71846878	0	1	0	1	1	1
510	-0.20928327	0.68721878	0	1	0	1	1	1
511	-0.20928327	0.68721878	0	1	0	1	1	1
512	-1.19879316	1.05531187	0	0	0	0	0	0
513	-1.69879316	1.10488610	0	0	0	0	0	0
514	-0.36890385	0.71846878	0	1	0	1	1	1
515	-0.48070657	0.71824575	0	1	0	1	1	1
516	-0.36250929	0.71824575	0	1	0	1	1	1
517	-0.32131409	0.71846878	0	1	0	1	1	1
518	-0.32131409	0.71846878	0	1	0	1	1	1
519	-0.68184973	0.76720265	1	1	0	0	0	1
520	-1.46365249	1.13629567	0	0	0	0	0	0
521	-1.21747277	1.05482449	0	0	0	0	0	0
522	-1.20258508	1.04540156	0	0	0	0	0	0
523	-1.22612214	1.06503743	0	0	0	0	0	0
524	-1.20653584	1.04700889	0	0	0	0	0	0
525	-1.19224167	1.06025426	0	0	0	0	0	0
526	-1.19399953	1.06092596	0	0	0	0	0	0
527	-1.19353093	1.13583619	0	0	0	0	0	0
528	-1.18862570	1.06330097	0	0	0	0	0	0
529	-0.53468988	0.69351177	0	0	0	0	0	0
530	-1.21637666	1.14273494	0	0	0	0	0	0

The Replacement Method (RM) procedure

The procedure of the RM technique is as follows: choose *d* descriptors, $\{X_1, X_2, \ldots, X_d\}$ at random and do a linear regression. Choose one of the descriptors of this set, called X_i, and replaced it by each of the *D* descriptors set (except itself) keeping the best resulting set. Since one can start replacing any of the *d* descriptors in the initial model, then a regression equation with *d* variables has *d* possible paths to achieve the final result; one example, the choice above will develop into path *i*. Next choose the variable with greatest relative error in it is coefficient (except the one replaced in the previous step) and replace it with all the D descriptors (except itself) keeping again the best set. Replace the entire remaining variable having greatest relative error in the coefficient and repeat the whole process. The process will repeat as many times as needed until the set of descriptors remains unchanged. At the end, we have the best model for the path *i*. Proceed in exactly the same way for all possible paths *i*=1, 2,…,*d*, compare the resulting models, and keep the best one. Our numerical experiments show that in this way one obtains a model almost as good as the best one with much less than *D!/(D-d)!d!* linear regressions when this combinatorial number is large.

The Balanced Subsets Method (BSM)

The BSM procedure for generating 3 balanced subsets (training, validation and test sets) consists on the next steps:

1. Prepare the $\mathbf{C}$ matrix that includes the experimental property of *N* compounds and *D* non-conformational descriptors. The size of $\mathbf{C}$ is $N \times (D+1)$.
2. Remove the linearly dependent descriptors from $\mathbf{C}$. The new size of $\mathbf{C}$ is $N \times (D^* + 1)$.
3. Standardize $\mathbf{C}$ for centering and scaling its matrix elements as the $\mathbf{C}^{\mathbf{stand}}$ matrix. This is done for discerning better the matrix elements. The size of $\mathbf{C}^{\mathbf{stand}}$ is $N \times (D^* + 1)$.
4. Use $\mathbf{C}^{\mathbf{stand}}$ together with the Euclidean metrics and $N_{runs} = 10,000$ runs in the optimization procedure of k-MCA, in order to achieve the best solution for creating $N^0_{train} = N_{train} - N_{\min\max}$ clusters, where N_{train} is the number of compounds in the training set and $N_{\min\max}$ is the number of compounds that have minimum or maximum values of the experimental property. This computes N^0_{train} cluster centroid locations as the $\mathbf{C}_1$ matrix of $N^0_{train} \times (D^* + 1)$ size.
5. The training set is prepared by selecting one compound per cluster which is nearer to the cluster centroid; the training set also includes the $N_{\min\max}$ compounds. In this way, the N_{train} compounds are identified.

6. Use $\mathbf{C}^{\mathbf{stand}}$ together with the Euclidean metrics and $N_{runs} = 10,000$ runs in the optimization procedure of k-MCA, in order to achieve the best solution for creating N_{val} clusters with the remaining $N - N_{train}$ compounds. This computes N_{val} cluster centroid locations as the $\mathbf{C}_2$ matrix of $N_{val} \times (D^* + 1)$ size.
7. The validation set is prepared by selecting one compound per cluster which is nearer to the cluster centroid. In this way, the N_{val} compounds are identified.
8. Finally, the test set is identified as the remaining $N_{test} = N - N_{train} - N_{val}$ compounds.
